# Pathogenic variants of ornithine transcarbamylase deficiency: Nation-wide study in Japan and literature review

**DOI:** 10.3389/fgene.2022.952467

**Published:** 2022-10-11

**Authors:** Jun Kido, Keishin Sugawara, Takaaki Sawada, Shirou Matsumoto, Kimitoshi Nakamura

**Affiliations:** ^1^ Department of Pediatrics, Kumamoto University Hospital, Kumamoto, Japan; ^2^ Department of Pediatrics, Faculty of Life Sciences, Kumamoto University, Kumamoto, Japan

**Keywords:** ornithine transcarbamylase deficiency, X-linked disorder, hyperammonemia, late onset OTCD, neonatal onset OTCD

## Abstract

Ornithine transcarbamylase deficiency (OTCD) is an X-linked disorder. Several male patients with OTCD suffer from severe hyperammonemic crisis in the neonatal period, whereas others develop late-onset manifestations, including hyperammonemic coma. Females with heterozygous pathogenic variants in the *OTC* gene may develop a variety of clinical manifestations, ranging from asymptomatic conditions to severe hyperammonemic attacks, owing to skewed lyonization. We reported the variants of *CPS1*, *ASS*, *ASL* and *OTC* detected in the patients with urea cycle disorders through a nation-wide survey in Japan. In this study, we updated the variant data of *OTC* in Japanese patients and acquired information regarding genetic variants of *OTC* from patients with OTCD through an extensive literature review. The 523 variants included 386 substitution (330 missense, 53 nonsense, and 3 silent), eight deletion, two duplication, one deletion-insertion, 55 frame shift, two extension, and 69 no category (1 regulatory and 68 splice site error) mutations. We observed a genotype–phenotype relation between the onset time (neonatal onset or late onset), the severity, and genetic mutation in male OTCD patients because the level of deactivation of *OTC* significantly depends on the pathogenic OTC variants. In conclusion, genetic information about *OTC* may help to predict long-term outcomes and determine specific treatment strategies, such as liver transplantation, in patients with OTCD.

## Introduction

Ornithine transcarbamylase (OTC; EC 2.1.3.3) is a mitochondrial enzyme that catalyzes the synthesis of citrulline from carbamoyl phosphate and ornithine during the urea cycle; inorganic phosphate is released as a by-product of the reaction. It is essential for the conversion of neurotoxic ammonia into non-toxic urea. In humans, OTC is exclusively expressed in the liver and small intestinal mucosa; however, it functions only in the liver during the urea cycle. The human *OTC* gene, which is 73 kb long and comprises 10 exons and nine introns (Hata et al., 1988), is located on the short arm of the X chromosome within band Xp21.1 (Lindgren et al., 1984). It encodes a precursor OTC protein that has a molecular weight of 39.9 kD and is composed of 354 amino acids. Upon entering the mitochondria, it undergoes post-transcriptional modification in which the 32 amino acid-long leader sequence is cleaved in two successive steps (Horwich et al., 1986). The mature OTC peptide has a molecular weight of 36.1 kD and is composed of 322 amino acids. The functional OTC holoenzyme is a homotrimer with a three-fold symmetry and three active sites, each of which is shared between two adjacent polypeptides (Shi et al., 1998).

The OTC deficiency (OTCD; MIM number: 311,250) is an X-linked disorder. Incidentally, the estimated frequency of OTCD is 1 per 80,000 births in Japan (Nagata et al., 1991), and recent studies indicate a prevalence of 1 per 62,000–77,000 births worldwide (Dionisi-Vici et al., 2002; Keskinen et al., 2008; Balasubramaniam et al., 2010; Summar et al., 2013). The OTCD phenotype is extremely heterogeneous. For instance, many male OTCD patients have severe hyperammonemic crisis in the neonatal stage, whereas others develop late-onset manifestations, including hyperammonemic coma (Kido et al., 2012; Kido et al., 2021a; Kido et al., 2021b). On the contrary, females with heterozygous pathogenic variants in the *OTC* gene may develop a variety of clinical manifestations, ranging from an asymptomatic condition to severe hyperammonemic attack, owing to the skewed lyonization phenomenon. Incidentally, the cloning of the human *OTC* gene has helped in the identification of mutations, most of which are “private” mutations (Yamaguchi et al., 2006). Majority of the mutation analysis may have been performed using PCR amplification of exons and flanking regions, followed by Sanger sequencing. In about 10%–15% of patients with clinically proven OTCD, no identifiable mutations have been detected in the routine molecular testing. In these patients, large deletions, duplications, and complex rearrangements associated with *OTC* or mutations in the promoter and enhancer region has been reported (Shchelochkov et al., 2009; Jang et al., 2018).

In the previous study (Kido et al., 2021d), we reported the variants of *CPS1*, *ASS*, *ASL,* and *OTC* detected in the patients with urea cycle disorders through a nation-wide survey in Japan and suggested that the onset time and severity in Japanese patients with OTCD can be estimated based on the type of *OTC* gene variant that they carry, thereby demonstrating a genotype–phenotype correlation in OTCD. In this study, we acquired information regarding 523 gene variants in patients with OTCD through a nationwide study in Japan and simultaneous literature review. Herein, we present our observations from the study and review. We also discuss the genotype–phenotype relationship and the clinical significance of these variants.

## Material and methods

Previously, we had conducted nation-wide surveys on Japanese patients with urea cycle disorders (UCDs), such as OTCD, carbamoyl phosphate synthetase 1 deficiency, N-acetylglutamate synthase deficiency, argininosuccinate synthetase deficiency, argininosuccinate lyase deficiency, and arginase 1 deficiency (Kido et al., 2021c; 2021a; 2021b; 2021d). In the current survey, we acquired the clinical data of 128 patients with OTCD (73 males and 55 females), including genetic information of 62 of them (57 families). These patients were diagnosed and/or treated in different departments, including pediatrics, neonatology, endocrinology and metabolism, genetics, and transplant surgery, from 78 different hospitals between January 2000 and March 2018. Additionally, we acquired the clinical data of patients diagnosed with OTCD in our institution as well.

As part of the literature review, we surveyed the genetic information of OTCD patients available on PubMed (https://pubmed.ncbi.nlm.nih.gov) or Google Scholar (https://scholar.google.com) using the keywords “*OTC* mutation” and “OTCD mutation.” Moreover, we surveyed variants in the *OTC* gene by quoting exact words/phrases/statements from related papers (Yamaguchi et al., 2006; Caldovic et al., 2015; Choi et al., 2015). We also evaluated variants of OTCD patients reported in 112 papers.

Variant nomenclature followed the guidelines established by the Human Genome Variation Society (http://varnomen.hgvs.org/) (den Dunnen et al., 2016), and the variants were categorized by protein level descriptions. The public database ClinVar (http://www.ncbi.nlm.nih.gov/clinvar) (Landrum et al., 2020) was used for the classification of each variant. Bioinformatic tools, PolyPhen-2 (http://genetics.bwh.harvard.edu/pph2) (Adzhubei et al., 2010) and SIFT (http://provean.jcvi.org/protein_batch_submit.php?species=human) (Choi et al., 2012) were used for predicting the potential impact of an amino acid alteration in missense mutations on the function of OTC.

### Ethics statement

This study was approved by the ethical committee of the Faculty of Life Science, Kumamoto University (Ethics. No.1527). All procedures followed were in accordance with the ethical standards of the responsible committee on human experimentation (institutional and national) and with the Helsinki Declaration of 1975, as revised in 2000. Informed consent was obtained from all patients or their legal guardians for being included in the study.

## Results

We acquired information regarding 523 genetic variants of OTCD patients through additional nation-wide survey conducted in Japan, as well as through a review of the existing relevant literature. These variants in the *OTC* gene included 386 substitution (330 missense, 53 nonsense, and 3 silent), eight deletion, two duplication, one deletion-insertion, 55 frame shift, two extension, and 69 no category (1 regulatory, 68 splice site error) mutations (Table 1; Supplementary data 1–3). Table 1 and Supplementary data 1 depicts the onset time of the OTCD symptoms and the maximum blood ammonia concentrations for each variant of the OTCD patients.

**TABLE 1:** Variants in the *OTC* gene and phenotype.

Variant no.	Nucleic acid	Amino acid	Location	Phenotype (onset-time)	NH3 (μmol/L)	References
Substitution (Missense variant)						
6	c.25T>G	p.Leu9*	Ex 1	N (2 days)	430	Kim et al. (2006)
12	c.67C>T	p.Arg23*	Ex 1	N (NA)	NA	Grompe et al. (1991)
				F (2 years)	NA	Matsuda and Tanase, (1997)
				F (2.5 years)	190	Lu et al. (2020)
				F (NA)	123	Kumar et al. (2021)
27	c.94C>T	p.Gln32*	Ex 2	F (15 m)	NA	Oppliger Leibundgut et al. (1997)
29	c.106C>T	p.Gln36*	Ex 2	F (NA)	NA	Genet et al. (2000)
57	c.148G>T	p.Gly50*	Ex 2	N (2 days)	1,700	Ali et al. (2018)
				F (8 m)	NA	Feldmann et al. (1992)
59	c.154G>T	p.Glu52*	Ex 2	F (NA)	NA	McCullough et al. (2000)
68	c.174G>A	p.Trp58*	Ex 2	N (NA)	NA	Yamaguchi et al. (2006)
				F (2 years)	NA	Lu et al. (2020)
77	c.205C>T	p.Gln69*	Ex 2	F (NA)	NA	Climent et al. (1999)
80	c.211G>T	p.Gly71*	Ex 2	F (9 m)	NA	Arranz et al. (2007)
85	c.219T>G	p.Tyr73*	Ex 3	N (NA)	477	Storkanova et al. (2013)
89	c.232C>T	p.Gln78*	Ex 3	N (NA)	NA	Yamaguchi et al. (2006)
94	c.245T>A	p.Leu82*	Ex 3	N (NA)	NA	Caldovic et al. (2015)
95	c.245T>G	p.Leu82*	Ex 3	F (NA)	NA	Tuchman et al. (2002)
96	c.245_246delTAinsAG	p.Leu82*	Ex 3	N (2 days)	789	Ali et al. (2018)
101	c.256dulT	p.Glu87*	Ex 3	N (NA)	NA	Caldovic et al. (2015)
109	c.274C>T	p.Arg92*	Ex 3	N (NA)	NA	Grompe et al. (1991)
				N (6 days)	879	Kim et al. (2006)
				N (NA)	1,200	Storkanova et al. (2013)
				F (NA)	NA	Gilbert-Dussardier et al. (1996)
135	c.313G>T	p.Gly105*	Ex 4	F (2y3m)	384	This study
142	c.327T>A	p.Cys109*	Ex 4	N (NA)	NA	Gobin-Limballe et al. (2021)
183	c.421C>T	p.Arg141*	Ex 5	N (6 days)	1,212	Matsuura et al. (1993)
				F (19 m)	NA	Hata et al. (1989)
						
				F (5 years)	183	Ogino et al. (2007)
				F (1 y)	575	Shao et al. (2017)
188	c.429T>A	p.Tyr143*	Ex 5	F (36 years)	280	Mukhtar et al. (2013)
189	c.430A>T	p.Lys144*	Ex 5	F (NA)	NA	Tuchman et al. (1995)
190	c.437C>G	p.Ser146*	Ex 5	N (NA)	NA	Genet et al. (2000)
198	c.460G>T	p.Glu154*	Ex 5	N (NA)	NA	Grompe et al. (1989)
218	c.491C>G	p.Ser164*	Ex 5	N (6 days)	NA	Hoshide et al. (1993)
				N (NA)	1,500	Storkanova et al. (2013)
				F (7 years)	NA	Matsuda and Tanase, (1997)
220	c.501C>A	p.Tyr167*	Ex 5	N (2 days)	NA	García-Pérez et al. (1995b)
221	c.501C>G	p.Tyr167*	Ex 5	N (2 days)	NA	Shimadzu et al. (1998)
248	c.538C>T	p.Gln180*	Ex 5	N (NA)	NA	Caldovic et al. (2015)
278	c.578G>A	p.Trp193*	Ex 6	N (7 days)	NA	Shimadzu et al. (1998)
				N (2 days)	>1,765	Ogino et al. (2007)
279	c.579G>A	p.Trp193*	Ex 6	N (20 days)	NA	Lu et al. (2020)
346	c.670G>T	p.Glu224*	Ex 7	NA	NA	Shchelochkov et al. (2009)
353	c.700G>T	p.Glu234*	Ex 7	N (NA)	NA	Yamaguchi et al. (2006)
354	c.703C>T	p.Gln235*	Ex 7	F (1.5 years)	350	Lu et al. (2020)
379	c.760A>T	p.Ala254*	Ex 8	F (NA)	NA	Caldovic et al. (2015)
381	c.766G>T	p.Gly256*	Ex 8	N/F (NA)	NA	Gobin-Limballe et al. (2021)
394	c.794G>A	p.Trp265*	Ex 8	L (4.3 years)	114	Lu et al. (2020)
396	c.795G>A	p.Trp265*	Ex 8	N (NA)	NA	Yamaguchi et al. (2006)
404	c.808C>T	p.Gln270*	Ex 8	N (NA)	NA	McCullough et al. (2000)
409	c.823A>T	p.Lys275*	Ex 8	N (4 days)	278	Kido et al. (2021c)
414	c.835C>T	p.Gln279*	Ex 8	N (NA)	NA	Tuchman et al. (2002)
417	c.852C>G	p.Tyr284*	Ex 8	F (14 m)	94	Wu et al. (2018)
419	c.853C>T	p.Gln285*	Ex 8	F (2 years)	489	Storkanova et al. (2013)
440	c.894G>A	p.Trp298*	Ex 9	F (11 m)	571	Kido et al. (2021d)
457	c.916A>T	p.Arg306*	Ex 9	NA	NA	Shchelochkov et al. (2009)
461	c.928G>T	p.Glu310*	Ex 9	N (3 days)	NA	Reish et al. (1993)
				F (3y5m)	118	Giorgi et al. (2000)
466	c.940G>T	p.Glu314*	Ex 9	F (2y4m)	166	Kido et al. (2021a)
475	c.958C>T	p.Arg320*	Ex 9	N (3 days)	782	Kim et al. (2006)
				L (6 m)	494	Kim et al. (2006)
				F (9 m)	494	Yoo et al. (1996)
				F (2 m)	NA	Matsuda and Tanase, (1997)
				F (2.3 years)	494	Choi et al. (2015)
477	c.962C>A	p.Ser321*	Ex 9	N (NA)	NA	Tuchman et al. (2002)
483	c.982G>T	p.Glu328*	Ex 9	N (NA)	NA	Yamaguchi et al. (2006)
485	c.988_990delAGAinsT	p.Arg330*	Ex 9	F (15 m)	NA	Climent and Rubio, (2002)
486	c.991A>T	p.Lys331*	Ex 9	F (NA)	NA	Yamaguchi et al. (2006)
488	c.995G>A	p.Trp332*	Ex 9	N (NA)	NA	Yamaguchi et al. (2006)
490	c.996G>A	p.Trp332*	Ex 9	N (2 days)	NA	Matsuura et al. (1994)
517	c.1042C>T	p.Gln348*	Ex 10	F (NA)	NA	Oppliger Leibundgut et al. (1997)
Substitution (Silent variant)						
335	c.663G>A	p.Lys221=	Ex 6	L (4 years)	NA	Shimadzu et al. (1998)
359	c.717G>A	p.Lys239=	Ex 7	F (NA)	NA	Tuchman et al. (1997)
423	c.867G>A	p.Lys289=	Ex 8	L (1 y)	4,500	Storkanova et al. (2013)
Deletion						
40	c.124_126del	p.Leu42del	Ex 2	F (1.1 y)	300	Lu et al. (2020)
41	c.126_128del	p.Leu43del	Ex 2	N (NA)	5,000	Storkanova et al. (2013)
93	c.243_245del	p.Leu82del	Ex 3	F (7 years)	248	Tuchman et al. (1995)
244	c.532_537del	p.Thr178_Leu179del	Ex 5	N (6 days)	NA	Shimadzu et al. (1998)
382	c.773_790del	p.Asn258_263del	Ex 8	NA	NA	Bijarnia-Mahay et al. (2018)
407	c.817_819del	p.Glu273del	Ex 8	L (NA)	NA	Ségues et al. (1996)
				L (1y3m)	218	Schultz and Salo, (2000)
				F (NA)	NA	Martín-Hernández et al. (2014)
460	c.928_930del	p.Glu310del	Ex 9	L (2 years)	200	Tuchman et al. (1995)
467	c.941_943del	p.Glu314del	Ex 9	F (NA)	NA	Yamaguchi et al. (2006)
Duplication						
170	c.390_392dup	p.Leu131dup	Ex 5	F (NA)	NA	Tuchman et al. (2002)
385	c.784_792dup	p.Thr262_Thr264dup	Ex 8	N (NA)	NA	Caldovic et al. (2015)
Deletion-insertion						
370	c.731_739del	p.Leu244_Thr247delinsPr	Ex 8	F (NA)	NA	Calvas et al. (1998)
Frame shift						
7	c.29_32del	p.Asn10Metfs*27	Ex 1	F (NA)	NA	Yamaguchi et al. (2006)
8	c.29dupA	p.Asn10Lysfs*6	Ex 1	F (NA)	NA	Yamaguchi et al. (2006)
10	c.42delT	p.Phe14Leufs*20	Ex 1	N (NA)	NA	Hwu et al. (2003b)
11	c.53delA	p.His18Profs*20	Ex 1	N (NA)	NA	Tuchman et al. (2002)
28	c.103insA	p.Val35Serfs*7	Ex 2	F (2 years)	580	Shao et al. (2017)
47	c.140delA	p.Asn47Thrfs*17	Ex 2	N (NA)	NA	Calvas et al. (1998)
				F (5.6 years)	500	Lu et al. (2020)
50	c.140dupA	p.Asn47Lysfs*8	Ex 2	N (2 days)	453	Kido et al. (2021b)
				F (NA)	NA	Yamaguchi et al. (2006)
51	c.140_141insG	p.Asn47Lysfs*8	Ex 2	N (3 days)	NA	Shimadzu et al. (1998)
54	c.144delT	p.Phe48Leufs*16	Ex 2	N (NA)	NA	Martín-Hernández et al. (2014)
78	c.207_226del	p.Gln69Hisfs*12	Ex 2	F (1.4 years)	197	Lu et al. (2020)
79	c.209_210del	p.Lys70Argfs*17	Ex 2	F (2y10 m)	344	Chongsrisawat et al. (2018)
108	c.271delA	p.Thr91Leufs*38	Ex 3	F (NA)	NA	Genet et al. (2000)
121	c.298delG	p.Gly100Alafs*21	Ex 3	N (NA)	NA	Gobin-Limballe et al. (2021)
144	c.330delT	p.Thr112Profs*9	Ex 4	N (NA)	NA	Calvas et al. (1998)
146	c.341_342del	p.Gln114Argfs*8	Ex 4	F (NA)	NA	Azevedo et al. (2006)
150	c.359_360del	p.Val120Glufs*2	Ex 4	F (NA)	NA	Yamaguchi et al. (2006)
151	c.364_365insTT	p.Glu122Valfs*66	Ex 4	F (NA)	NA	Yamaguchi et al. (2006)
155	c.376delG	p.Asp126Thrfs*61	Ex 4	F (NA)	NA	Yamaguchi et al. (2006)
171	c.391_397dup	p.Ser133Ilefs*3	Ex 5	F (22 m)	NA	Arranz et al. (2007)
175	c.403delG	p.Ala135Glnfs*52	Ex 5	N (4 days)	3,000	Tuchman et al. (1992)
195	c.451delC	p.Leu151Trpfs*36	Ex 5	F (NA)	NA	Tuchman et al. (2002)
199	c.461_471del	p.Glu154Alafs*18	Ex 5	N (NA)	1,200	Storkanova et al. (2013)
233	c.516_525del	p.Leu173Thrfs*11	Ex 5	N (NA)	NA	Arranz et al. (2007)
236	c.523_536del	p.Asp175Profs*5	Ex 5	F (NA)	335	Kido et al. (2021c)
242	c.530_533dup	p.Leu179Hisfs*7	Ex 5	N (4 days)	NA	Gilbert-Dussardier et al. (1996)
263	c.552insGAAC	p.Ser185Efs*41	Ex 6	F (2.4 years)	385	Lu et al. (2020)
265	c.561delA	p.Gly188Valfs*18	Ex 6	F (NA)	NA	Caldovic et al. (2015)
266	c.562_563del	p.Gly188Serfs*36	Ex 6	NA	NA	Shchelochkov et al. (2009)
270	c.568delA	p.Thr190Profs*16	Ex 6	NA	NA	Shchelochkov et al. (2009)
271	c.568dupA	p.Thr190Asnfs*35	Ex 6	F (NA)	NA	Gobin-Limballe et al. (2021)
272	c.571delC	p.Leu191Serfs*15	Ex 6	N (7 days)	860	Kim et al. (2006)
285	c.586delG	p.Asp196Metfs*10	Ex 6	F (18 m)	NA	Climent and Rubio, (2002)
298	c.597_598del	p.Ile200Profs*24	Ex 6	N (NA)	NA	Tuchman et al. (1994)
318	c.630delA	p.Lys210Asnfs*20	Ex 6	F (NA)	NA	Martín-Hernández et al. (2014)
326	c.645dupT	p.Gln216Serfs*9	Ex 6	N (NA)	NA	Tuchman et al. (1994)
345	c.664_667delinsAC	p.Gly222Thrfs*2	Ex 7	N (3 days)	1,000	Lee et al. (2014)
				F (0.8 years)	233	Choi et al. (2015)
351	c.697delG	p.Leu232Leufs*14	Ex 7	N (NA)	NA	Laróvere et al. (2018)
378	c.759delA	p.Ala254Argfs*7	Ex 8	N (NA)	NA	Yamaguchi et al. (2006)
397	c.796_805del	p.Ile265_Gly268delinsAspfs*19	Ex 8	N (6 days)	639	Kim et al. (2006)
				N (6 days)	639	Choi et al. (2015)
399	c.799_800insA	p.Ser267Lysfs*26	Ex 8	F (12.8 years)	307	Choi et al. (2015)
406	c.813_814delAGinsC	p.Glu271Aspfs*28	Ex 8	N (2 days)	NA	Khoo et al. (1999)
				F (1 y)	560	Ali et al. (2018)
408	c.818delA	p.Glu273Glyfs*16	Ex 8	N (NA)	NA	Yamaguchi et al. (2006)
413	c.834_840del	p.Gln279Serfs*8	Ex 8	L (18 m)	256	Lee et al. (2018)
				L (1y6m)	256	Kido et al. (2021d)
418	c.853delC	p.Gln285Argfs*4	Ex 8	N (3 days)	856	Kim et al. (2006)
422	c.861_862insAC	p.Met288Thrfs*2	Ex 8	F (NA)	NA	Caldovic et al. (2015)
432	c.876delA	p.Val293Leufs*30	Ex 9	N (NA)	NA	Yamaguchi et al. (2006)
433	c.882delT	p.Ala295Profs*28	Ex 9	N (3 days)	NA	Reish et al. (1993)
434	c.888delT	p.Asp297Thrfs*26	Ex 9	F (1.5 years)	96	Bernal et al. (2021)
436	c.890_893del	p.Asp297Glyfs*25	Ex 9	N (53 h)	>1,000	Yamanouchi et al. (2002)
437	c.892_893del	p.Trp298Aspfs*15	Ex 9	F (24 years)	23.5	Schimanski et al. (1996)
446	c.906delC	p.Cys303Alafs*20	Ex 9	F (NA)	NA	Yamaguchi et al. (2006)
462	c.929_931del	p.Glu310Valfs*45	Ex 9	L (6 m)	396	Storkanova et al. (2013)
				L (2.1 y)	257	Lu et al. (2020)
				L (11 m)	105	Kido et al. (2021a)
481	c.970_979del	p.Phe324Glnfs*16	Ex 9	5 days (M)	461	Wang et al. (2022b)
518	c.1043delA	p.Gln348Argfs*47	Ex 10	F (2 years)	76	Storkanova et al. (2013)
520	c.1052delA	p.Lys351Serfs*44	Ex 10	F (NA)	NA	Gobin-Limballe et al. (2021)
Extension						
522	c.1063T>C	p.*355Argext*15	Ex 10	F (NA)	NA	Caldovic et al. (2015)
523	c.1065A>T	p.*355Cysext*15	Ex 10	L (2 years)	499	Storkanova et al. (2013)

N, neonatal-onset; L, late-onset; F, female; NA, not available; mo, mosaicism.

The variants were categorized by protein level descriptions.

Among the missense variants, 108 variants have been identified in the male patients with neonatal onset of OTCD, while 81 variants have been identified in the male patients with late onset of OTCD. Eleven variants, namely the c.119G>A (p.Arg40His), c.304G>C (p.Ala102Pro), c.386G>A (p.Arg129His), c.481A>G (p.Asn161Asp), c.535C>T (p.Leu179Phe), c.540G>C (p.Gln180His), c.562G>C (p.Gly188Arg), c.725C>T (p.Thr242Ile), c.803T>C (p.Met268Thr), c.829C>T (p.Arg177Trp), and c.1028C>G (p.Thr343Arg), have been identified in case of both neonatal and late onset male OTCD patients. Additionally, the c.128T>C (p.Leu43Pro), c.530T>G (p.Leu177Arg), c.628A>C (p.Lys210Gln), and c.1025T>G (p.Leu342Pro) variants have been identified in female patients with neonatal onset of OTCD.

All nonsense variants detected in the male OTCD patients have been identified as the neonatal-onset type variant. Additionally, two silent variants, namely c.663G>A (p.Lys221=) and c.867G>A (p.Lys289=); three frame shift variants, specifically c.834_840delCCAGGCT (p.Gln279Serfs*8), c.929_931delAAG (p.Glu310Valfs*45), and c.1065A>T (p.*355Cysext*14); and two deletion/duplication variants, namely c.817_819delGAG (p.Glu273del) and c.928_930delGAA (p.Glu310del), have been identified in the late onset male OTCD patients.

The splicing-disrupting variants in introns 2, 3, 8, and 9 have been identified in case of both neonatal and late onset male OTCD patients. All splicing-disrupting variants in introns 5, 6, and 7 have been identified in the male patients with neonatal onset of OTCD. Moreover, although majority of the splicing-disrupting variants identified in introns 1 and 4 are specific for the male patients with neonatal onset of OTCD, only three variants, namely c.78-2A>G variant in intron 1 and c.386 + 1G>T, as well as c.386+4delT in intron 4, are specific for the late onset male OTCD patients.

**FIGURE 1:**
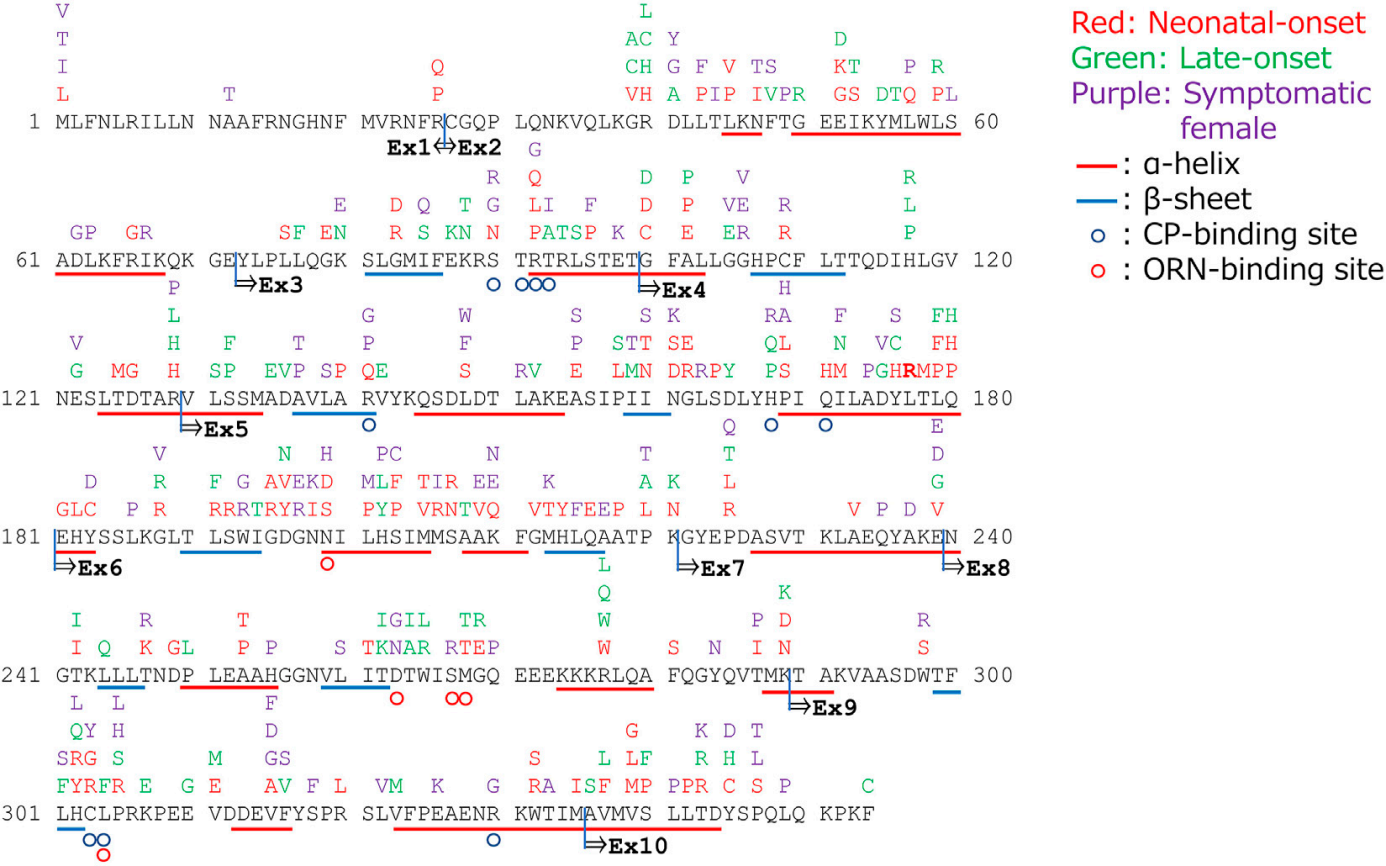
Amino acid substitutions in the gene encoding ornithine transcarbamylase (*OTC*) and their correlated phenotypes.

Figure 1 demonstrates the amino acid substitutions in the *OTC* gene as detected in patients with OTCD. Incidentally, amino acid substitutions in exons 5 or 6 and in the α-helix or *β*-sheet structures are likely to result in neonatal onset of OTCD. Moreover, amino acid substitutions in positions 40, 52, 53, 59, 100, 102, 129, 158, 172, 176, 179, 180, 188, 191, 196, 220, 221, 225, 239, 242, 268, 269, 277, 289, 302,305,311, 337, 340, 343, and 345 are related to both neonatal and late onset OTCD patients.

## Discussion

In this study, we have suggested the genotype–phenotype correlations about the onset time of OTCD symptoms and the maximum blood ammonia levels, with respect to the identified variants of the *OTC* gene. While we could not demonstrate a linear relationship between genetic mutation, protein activity quantification, and clinical morbidity, we revealed the impact of the amino acid substitutions in OTC on the time of onset of the symptoms. According to Tuchman’s study, among the gene mutations leading to OTCD, majority (approximately 84%) are single-base substitutions, while small deletions or insertions and large deletions comprise a smaller proportion of the mutations (12% and 4%, respectively). The mutations are largely “private,” with recurrent mutations occurring mainly in CpG dinucleotides (Tuchman et al., 2002). Therefore, these are known as the mutation hotspots. Incidentally, a previous study indicated that majority of the mutations (80%) arise in the male germ cells (Tuchman et al., 1998). However, our survey demonstrated that the variants are equally likely to arise in any exon of the *OTC* gene.

The functional OTC holoenzyme is a homotrimeric protein, and each subunit contains an N-terminal domain that binds to carbamoyl phosphate and a C-terminal domain that binds to L-ornithine (Shi et al., 1998). Therefore, these domains are essential for the formation of the enzyme’s active site. Moreover, the α-helix and the β-sheet conformations are essential for retaining the structure of the functional enzyme. Hence, OTC variants that cannot retain the enzyme structure lead to the neonatal onset of OTCD, even if it is an amino acid substitution variant. Moreover, amino acids substitutions in the same position could lead to both neonatal and late onset of OTCD. The time of onset of disease symptoms and the disease severity may vary since the homotrimeric arrangement of the functional protein depends on the condition in the body. Splicing-disrupting mutations in the introns lead to heterogeneous variants, which, in turn, may be influenced by the condition in the body (Olga et al., 2020); hence, the OTC proteins synthesized are not all abnormal. Majority of the splicing-disrupting variants in intron 4 and all the splicing-disrupting variants in introns 5, 6, and 7 were associated with neonatal onset of OTCD. Although the number of exon sites removed in each splicing-disrupting variant was not evaluated, exons 5, 6, 7, and 8 were speculated to be essential for maintaining OTC function.

Neonatal onset of OTCD leads to severe symptoms, and a majority of these patients suffer from hyperammonemia attacks resulting in a maximum blood ammonia concentration of ≥360 μmol/L at the time of onset (Kido et al., 2018; 2021b; 2021a). Such hyperammonemia attacks could damage the brain significantly and lead to poor neurodevelopmental outcomes in patients with OTCD (Kido et al., 2012; 2021a; 2021b).

Family members of OTCD patients, males as well as females, may also develop symptoms of OTCD, such as hyperammonemia attacks. Incidentally, if a male child is born to a female who has a family history of OTCD and possesses a known neonatal onset type variant, then immediate intervention will be necessary after birth to prevent a hyperammonemia attack that may cause blood ammonia levels to rise above 360 μmol/L. In fact, if the maximum blood ammonia levels can be controlled within 360 μmol/L during the first as well as subsequent hyperammonemia attacks, then these patients with neonatal onset OTCD are likely to acquire normal neurodevelopmental outcomes. Moreover, if the maximum ammonia concentrations could be controlled within 360 μmol/L in patients with neonatal onset OTCD, then early liver transplantation may help to achieve a stable overall health condition as well as proper neurodevelopmental outcomes (Kido et al., 2018; 2021a). Such patients may live a life with normal social activity.

There is a degree of genotype–phenotype correlation in male OTCD patients because the level of deactivation of *OTC* depends extensively on the pathogenic *OTC* variants. Therefore, the information about the *OTC* variants discussed in this study may help to develop early intervention strategies for patients who possess variants associated with neonatal onset OTCD; early liver transplantation should be considered as an optional therapy for such patients. Other notable OTC therapeutic options include gene and exon skipping therapy that may become available for clinical application in the near future (Supplementary data 4) (Balestra et al., 2020; Baruteau et al., 2021; and Wang et al., 2012 and, 2022a).

In future, it is important to establish a new medical system that will be able to provide a better prognosis by referring to the patient’s genetic information and intervening at an appropriate time. Moreover, we should consider the need of more comprehensive prenatal genetic testing system for *OTC* gene because the current prenatal genetic testing of *OTC* is applied to known mutations in the families with *OTC* gene mutation or OTCD patients in each institution in Japan. These will help to develop subsequent treatment strategies, including liver transplantation, which may help to save the patients’ lives.

In conclusion, we investigated the impact of OTCD variants on clinical aspects of Japanese patients through an additional nationwide study and an extensive literature review. Genetic information about *OTC* variations may help to predict long-term outcomes of the OTCD patients, as well as determine specific treatment strategies, such as liver transplantation. In particular, such genetic information is beneficial for performing prenatal diagnosis and designing intervention strategies for neonates born to females possessing the neonate onset variants.

## Data Availability

The datasets presented in this study can be found in online repositories. The names of the repository/repositories and accession number(s) can be found in the article/Supplementary Material.
