# Supplementary figures and images for: Pathogenic variants of ornithine transcarbamylase deficiency: Nation-wide study in Japan and literature review
Source: Front Genet. 2022 Oct 11;13:952467. doi: 10.3389/fgene.2022.952467 (PMC9593096; doi:10.3389/fgene.2022.952467)

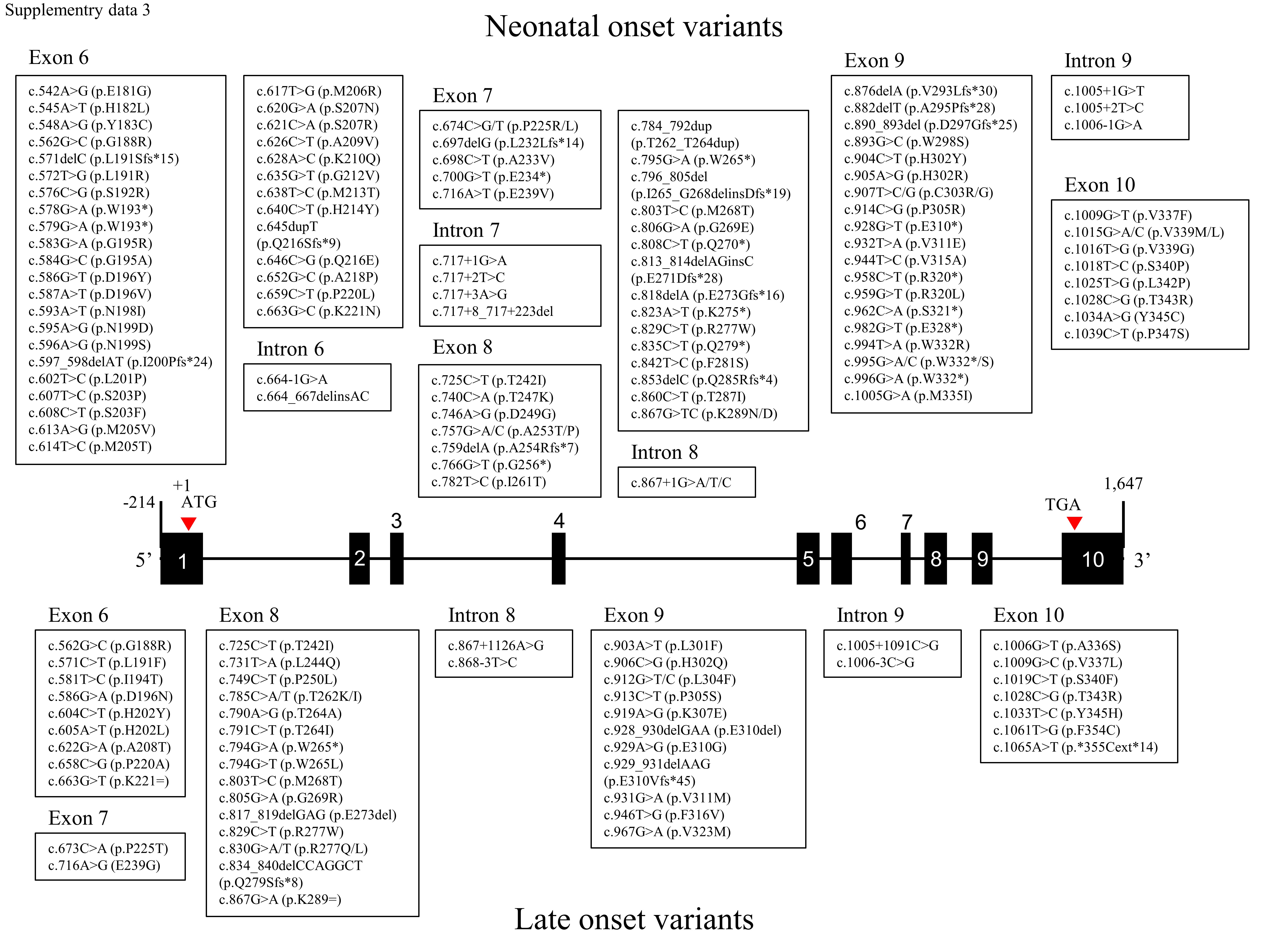

Supplement: Supplementary file 1 [file Image2.jpg]

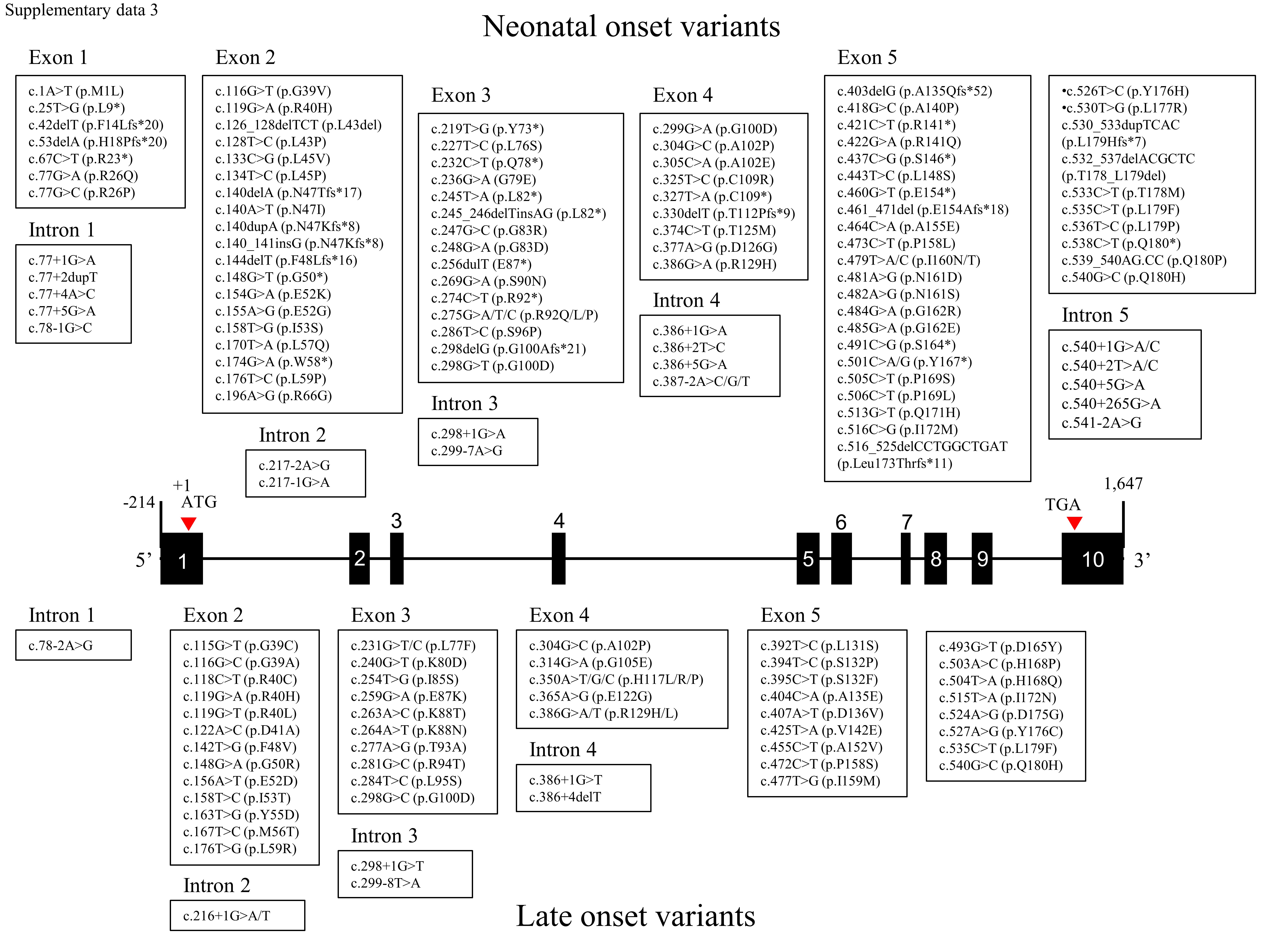

Supplement: Supplementary file 4 [file Image1.jpg]
