# Supplementary material for: Pathogenic variants of ornithine transcarbamylase deficiency: Nation-wide study in Japan and literature review
Source: Front Genet. 2022 Oct 11;13:952467. doi: 10.3389/fgene.2022.952467 (PMC9593096; doi:10.3389/fgene.2022.952467)
Supplement: Supplementary file 2 [file DataSheet3.docx]

Supplementary data 3. Gene therapy-based clinical trials for OTCD

| Disease | NCT number | Sponsor (Code name) | Status* | n (Age/Sex) | Vector (carrying gene), Administration  Adverse events (Ads) | References |
| --- | --- | --- | --- | --- | --- | --- |
| OTC deficiency | 05345171 | Ultragenyx Pharmaceutical Inc (DTX301; avalotcagene ontaparvovec) | Ph 3, Recruiting | 50 (>12y/All) | AAV8 (OTC), Intravenous | Wang et al. (2012 and 2022) |
|  | 03636438 |  | LT, Active, not recruiting | 11 (>18y/All) |  |  |
|  | 02991144 |  | Ph 1/2, Completed |  |  |  |
|  | 00004498 | University of Pennsylvania | Ph 1, Terminated | 3 (18-69y/All) | AdV (OTC), Intravascularly |  |
|  | 00004386 | Eunice Kennedy Shriver National Institute of Child Health and Human Development (NICHD) | Ph 1, Terminated | 3 (18-65y/All) | AdV (OTC), Infused into the liver |  |

LT: long-term follow-up study
