# Supplementary material for: Pathogenic variants of ornithine transcarbamylase deficiency: Nation-wide study in Japan and literature review
Source: Front Genet. 2022 Oct 11;13:952467. doi: 10.3389/fgene.2022.952467 (PMC9593096; doi:10.3389/fgene.2022.952467)
Supplement: Supplementary file 3 [file DataSheet2.docx]

Supplementary data 2. Variants in the *OTC* gene

| No. | dbSNP | Nucleic acid | Amino acid | Location | ClinVar | Polyphen-2 (Score) | SIFT (Score) | References |
| --- | --- | --- | --- | --- | --- | --- | --- | --- |
| 1 | rs191615506 | c.-366A>G | - | 5’UTR | Benign/Likely benign | - | - | Luksan (2010 |
| 2 | rs67752076 | c.1A>G | p.Met1Val | Exon 1 | pathogenic | benign (0.064) | Tolerated (0.114) | Oppliger Leibundgut (1997) |
| 3 | rs67752076 | c.1A>T | p.Met1Leu | Exon 1 | pathogenic | benign (0.141) | Damaging (0.029) | Yamaguchi (2006) |
| 4 | rs72552295 | c.2T>C | p.Met1Thr | Exon 1 | pathogenic | possibly damaging (0.895) | Damaging (0.005) | Yamaguchi (2006) |
| 5 | rs72552296 | c.3G>A | p.Met1Ile | Exon 1 | pathogenic | possibly damaging (0.465) | Damaging (0.015) | Climent (2002), Lu (2020) |
| 6 |  | c.25T>G | p.Leu9* | Exon 1 | NR | - | - | Kim (2006) |
| 7 | rs72552297 | c.29_32del | p.Asn10Metfs*27 | Exon 1 | pathogenic | - | - | Yamaguchi (2006) |
| 8 |  | c.29dupA | p.Asn10Lysfs*6 | Exon 1 | NR | - | - | Yamaguchi (2006) |
| 9 |  | c.34G>A | p.Ala12Thr | Exon 1 | NR | benign (0.000) | Tolerated (0.148) | Tanaka (2005) |
| 10 | rs72552298 | c.42delT | p.Phe14Leufs*20 | Exon 1 | pathogenic | - | - | Hwu (2003b) |
| 11 | rs72552299 | c.53delA | p.His18Profs*20 | Exon 1 | pathogenic | - | - | Tuchman (2002) |
| 12 | rs72552300 | c.67C>T | p.Arg23* | Exon 1 | pathogenic | - | - | Grompe (1991), Matsuda (1997), Lu (2020), Kumar (2021) |
| 13 | rs68031618 | c.77G>A | p.Arg26Gln | Exon 1 | pathogenic | benign (0.002) | Tolerated (0.323) | Grompe (1989), Kim (2006), Choi (2015), Shao (2017), Zheng (2020), Kido (2021) |
| 14 | rs68031618 | c.77G>C | p.Arg26Pro | Exon 1 | pathogenic | benign (0.123) | Tolerated (0.119) | Yamaguchi (2006), Kim (2006), Choi (2015) |
| 15 | rs67077695 | c.77+1G>A | - | Intron 1 | pathogenic | - | - | Matsuda (1997, Yamaguchi (2006) |
| 16 | rs67077695 | c.77+1G>T | - | Intron 1 | pathogenic | - | - | Tuchman (1997, This study |
| 17 |  | c.77+1G>C | - | Intron 1 | NR | - | - | Lu (2020) |
| 18 | rs1569270890 | c.77+2dupT | - | Intron 1 | likely pathogenic | - | - | Yamaguchi (2006) |
| 19 | rs72558496 | c.77+3_77+6del | - | Intron 1 | pathogenic | - | - | Tuchman (2002) |
| 20 | rs72552301 | c.77+4A>C | - | Intron 1 | pathogenic | - | - | Hoshide (1996) |
| 21 | rs72552302 | c.77+5G>A | - | Intron 1 | pathogenic | - | - | Tuchman (1997) |
| 22 |  | c.77+5G>C | - | Intron 1 | pathogenic | - | - | Gobin-Limballe (2021) |
| 23 | rs72554303 | c.78-3C>G | - | Intron 1 | pathogenic | - | - | Bisanzi (2002) |
| 24 |  | c.78-2A>G | - | Intron 1 | NR | - | - | Lu (2020) |
| 25 |  | c.78-1G>A | - | Intron 1 | NR | - | - | Olga (2020) |
| 26 | rs281865554 | c.78-1G>C | - | Intron 1 | pathogenic | - | - | Yamaguchi (2006) |
| 27 | rs72554304 | c.94C>T | p.Gln32* | Exon 2 | pathogenic | - | - | Oppliger Leibundgut (1997) |
| 28 |  | c.103insA | p.Val35Serfs*7 | Exon 2 | NR | - | - | Shao (2017) |
| 29 | rs72554305 | c.106C>T | p.Gln36* | Exon 2 | pathogenic | - | - | Genet (2000) |
| 30 | rs72554306 | c.115G>T | p.Gly39Cys | Exon 2 | pathogenic | probably damaging (1.000) | Damaging (0.015) | Calvas (1998) |
| 31 | rs1602014500 | c.116G>A | p.Gly39Asp | Exon 2 | uncertain significance | probably damaging (1.000) | Damaging (0.034) | Shchelochkov (2009) |
| 32 |  | c.116G>T | p.Gly39Val | Exon 2 | NR | probably damaging (1.000) | Damaging (0.024) | Zheng (2020) |
| 33 |  | c.116G>C | p.Gly39Ala | Exon 2 | pathogenic | probably damaging (0.993) | Tolerated (0.194) | Gobin-Limballe (2021) |
| 34 | rs72554307 | c.118C>T | p.Arg40Cys | Exon 2 | Pathogenic/likely pathogenic | probably damaging (1.000) | Damaging (0.009) | Oppliger Leibundgut (1995) |
| 35 | rs72554308 | c.119G>A | p.Arg40His | Exon 2 | pathogenic | possibly damaging (0.668) | Damaging (0.018) | Tuchman (1994, Nishiyori (1997), Takanashi (2002), Cavicchi (2014), Shao (2017), Zhou (2020), Lu (2020), Kido (2021), This study |
| 36 |  | c.119G>T | p.Arg40Leu | Exon 2 | NR | probably damaging (1.000) | Damaging (0.003) | Cavicchi (2014) |
| 37 |  | c.121G>T | p.Asp41Tyr | Exon 2 | NR | possibly damaging (0.735) | Damaging (0.009) | Lee (2020) |
| 38 | rs74518351 | c.122A>G | p.Asp41Gly | Exon 2 | likely pathogenic | possibly damaging (0.485) | Tolerated (0.057) | Yamaguchi (2006) |
| 39 |  | c.122A>C | p.Asp41Ala | Exon 2 | NR | benign (0.336) | Damaging (0.016) | Lu (2020) |
| 40 |  | c.124_126del | p.Leu42del | Exon 2 | NR | - | - | Lu (2020) |
| 41 |  | c.126_128del | p.Leu43del | Exon 2 | NR | - | - | Storkanova (2013) |
| 42 | rs72554309 | c.127C>T | p.Leu43Phe | Exon 2 | pathogenic | probably damaging (1.000) | Damaging (0.003) | Oppliger Leibundgut (1997) |
| 43 |  | c.128T>C | p.Leu43Pro | Exon 2 | pathogenic | probably damaging (1.000) | Damaging (0.001) | Gobin-Limballe (2021) |
| 44 | rs72554310 | c.131C>T | p.Thr44Ile | Exon 2 | pathogenic | probably damaging (0.995) | Damaging (0.002) | Yoo (1996), Kim (2006) |
| 45 | rs72554311 | c.133C>G | p.Leu45Val | Exon 2 | pathogenic | probably damaging (0.981) | Tolerated (0.054) | Tuchman (1998), Ali (2018) |
| 46 | rs72554312 | c.134T>C | p.Leu45Pro | Exon 2 | pathogenic | probably damaging (1.000) | Damaging (0.001) | Grompe (1989) |
| 47 | rs72554313 | c.140delA | p.Asn47Thrfs*17 | Exon 2 | pathogenic | - | - | Calvas (1998), Lu (2020) |
| 48 | rs67939655 | c.140A>C | p.Asn47Thr | Exon 2 | uncertain significance | benign (0.041) | Damaging (0.005) | Yamaguchi (2006) |
| 49 | rs67939655 | c.140A>T | p.Asn47Ile | Exon 2 | pathogenic | possibly damaging (0.477) | Damaging (0.001) | Tuchman (1997), Choi (2015), Kido (2021) |
| 50 |  | c.140dupA | p.Asn47Lysfs*8 | Exon 2 | NR | - | - | Yamaguchi (2006), Kido (2021) |
| 51 | rs72554314 | c.140_141insG | p.Asn47Lysfs*8 | Exon 2 | likely pathogenic | - | - | Shimadzu (1998) |
| 52 |  | c.142T>G | p.Phe48Val | Exon 2 | NR | possibly damaging (0.883) | Tolerated (0.051) | Kido (2021) |
| 53 | rs72554315 | c.143T>C | p.Phe48Ser | Exon 2 | pathogenic | probably damaging (1.000) | Damaging (0.002) | Genet (2000) |
| 54 |  | c.144delT | p.Phe48Leufs*16 | Exon 2 | likely pathogenic | - | - | Martín-Hernández (2014) |
| 55 | rs72554316 | c.145A>C | p.Thr49Pro | Exon 2 | pathogenic | probably damaging (0.998) | Damaging (0.002) | Yamaguchi (2006) |
| 56 | rs67486158 | c.148G>A | p.Gly50Arg | Exon 2 | pathogenic | possibly damaging (0.817) | Tolerated (0.242) | Tuchman (1997) |
| 57 | rs67486158 | c.148G>T | p.Gly50* | Exon 2 | pathogenic | - | - | Feldmann (1992), Ali (2018) |
| 58 | rs66521141 | c.154G>A | p.Glu52Lys | Exon 2 | pathogenic | probably damaging (1.000) | Damaging (0.002) | McCullough (2000), Martín-Hernández (2014) |
| 59 | rs66521141 | c.154G>T | p.Glu52* | Exon 2 | pathogenic | - | - | McCullough (2000) |
| 60 | rs72554317 | c.155A>G | p.Glu52Gly | Exon 2 | pathogenic | probably damaging (1.000) | Damaging (0.001) | Yamaguchi (2006) |
| 61 | rs72554318 | c.156A>T | p.Glu52Asp | Exon 2 | pathogenic | probably damaging (1.000) | Damaging (0.004) | McCullough (2000) |
| 62 | rs66677059 | c.158T>C | p.Ile53Thr | Exon 2 | pathogenic | probably damaging (1.000) | Damaging (0.004) | Yamaguchi (2006) |
| 63 | rs66677059 | c.158T>G | p.Ile53Ser | Exon 2 | pathogenic | probably damaging (1.000) | Damaging (0.002) | Tuchman (2002) |
| 64 | rs72554319 | c.163T>G | p.Tyr55Asp | Exon 2 | pathogenic | probably damaging (0.995) | Tolerated (0.487) | Nishiyori (1998) |
| 65 | rs72554320 | c.167T>C | p.Met56Thr | Exon 2 | likely pathogenic | possibly damaging (0.524) | Damaging (0.011) | Tuchman (1997) |
| 66 | rs72554321 | c.170T>A | p.Leu57Gln | Exon 2 | pathogenic | probably damaging (1.000) | Damaging (0.001) | Yamaguchi (2006) |
| 67 |  | c.170T>C | p.Leu57Pro | Exon 2 | NR | probably damaging (1.000) | Damaging (0.001) | Choi (2015) |
| 68 | rs72554322 | c.174G>A | p.Trp58* | Exon 2 | pathogenic | - | - | Yamaguchi (2006), Lu (2020) |
| 69 |  | c.176T>C | p.Leu59Pro | Exon 2 | NR | probably damaging (0.995) | Tolerated (0.274) | Zhou (2020) |
| 70 |  | c.176T>G | p.Leu59Arg | Exon 2 | NR | benign (0.254) | Tolerated (0.535) | Azevedo (2006) |
| 71 | rs72554323 | c.179C>T | p.Ser60Leu | Exon 2 | pathogenic | possibly damaging (0.916) | Damaging (0.001) | Tuchman (1995a), Kido (2021) |
| 72 |  | c.184G>C | p.Asp62His | Exon 2 | NR | possibly damaging (0.794) | Tolerated (0.101) | Shchelochkov (2009) |
| 73 |  | c.185A>G | p.Asp62Gly | Exon 2 | NR | probably damaging (0.998) | Tolerated (0.130) | Caldovic (2015) |
| 74 | rs72554324 | c.188T>C | p.Leu63Pro | Exon 2 | pathogenic | probably damaging (1.000) | Tolerated (0.055) | Oppliger Leibundgut (1997) |
| 75 |  | c.196A>G | p.Arg66Gly | Exon 2 | NR | probably damaging (1.000) | Tolerated (0.106) | Kido (2021) |
| 76 | rs72554325 | c.200T>G | p.Ile67Arg | Exon 2 | pathogenic | probably damaging (0.999) | Damaging (0.015) | Yamaguchi (2006) |
| 77 | rs72554326 | c.205C>T | p.Gln69* | Exon 2 | pathogenic | - | - | Climent (1999) |
| 78 |  | c.207_226del | p.Gln69Hisfs*12 | Exon 2 | NR | - | - | Lu (2020) |
| 79 |  | c.209_210del | p.Lys70Argfs*17 | Exon 2 | NR | - | - | Chongsrisawat (2018) |
| 80 |  | c.211G>T | p.Gly71* | Exon 2 | NR | - | - | Arranz (2007) |
| 81 | rs66693137 | c.216+1G>A | - | Intron 2 | pathogenic | - | - | Oppliger Leibundgut (1996b), Kido (2021) |
| 82 | rs66693137 | c.216+1G>T | - | Intron 2 | pathogenic | - | - | Azevedo (2002) |
| 83 |  | c.217-2A>G | - | Intron 2 | pathogenic | - | - | Gobin-Limballe (2021) |
| 84 | rs72554327 | c.217-1G>A | - | Intron 2 | pathogenic | - | - | Tuchman (1992) |
| 85 |  | c.219T>G | p.Tyr73* | Exon 3 | NR | - | - | Storkanova (2013) |
| 86 | rs72554328 | c.227T>C | p.Leu76Ser | Exon 3 | pathogenic | probably damaging (1.000) | Tolerated (0.880) | Genet (2000), Kido (2021) |
| 87 | rs72554329 | c.231G>T | p.Leu77Phe | Exon 3 | pathogenic | probably damaging (0.995) | Damaging (0.002) | McCullough (2000) |
| 88 |  | c.231G>C | p.Leu77Phe | Exon 3 | NR | probably damaging (0.995) | Damaging (0.002) | Shao (2017) |
| 89 | rs72554330 | c.232C>T | p.Gln78* | Exon 3 | pathogenic | - | - | Yamaguchi (2006) |
| 90 | rs72554331 | c.236G>A | p.Gly79Glu | Exon 3 | pathogenic | probably damaging (1.000) | Damaging (0.002) | Tuchman (1992) |
| 91 | rs72554332 | c.238A>G | p.Lys80Glu | Exon 3 | pathogenic | possibly damaging (0.736) | Damaging (0.025) | Schultz (2000) |
| 92 | rs72554333 | c.240G>T | p.Lys80Asn | Exon 3 | pathogenic | probably damaging (0.999) | Damaging (0.002) | Galloway (2000) |
| 93 | rs72554334 | c.243_245del | p.Leu82del | Exon 3 | pathogenic | - | - | Tuchman (1995b) |
| 94 |  | c.245T>A | p.Leu82* | Exon 3 | NR | - | - | Caldovic (2015) |
| 95 | rs72554335 | c.245T>G | p.Leu82* | Exon 3 | pathogenic | - | - | Tuchman (2002) |
| 96 |  | c.245_246delTAinsAG | p.Leu82* | Exon 3 | NR | - | - | Ali (2018) |
| 97 | rs72554336 | c.247G>C | p.Gly83Arg | Exon 3 | pathogenic | benign (0.065) | Damaging (0.001) | Tuchman (1997) |
| 98 | rs72554337 | c.248G>A | p.Gly83Asp | Exon 3 | pathogenic | possibly damaging (0.457) | Damaging (0.001) | Bartholomew (1998) |
| 99 |  | c.254T>A | p.Ieu85Gln | Exon 3 | NR | probably damaging (1.000) | Damaging (0.001) | Kido (2021) |
| 100 |  | c.254T>G | p.Ile85Ser | Exon 3 | NR | probably damaging (1.000) | Damaging (0.001) | Martín-Hernández (2014) |
| 101 |  | c.256dulT | p.Glu87* | Exon 3 | NR | - | - | Caldovic (2015) |
| 102 | rs72554338 | c.259G>A | p.Glu87Lys | Exon 3 | pathogenic | possibly damaging (0.881) | Tolerated (0.063) | Tuchman (1995a) |
| 103 |  | c.263A>C | p.Lys88Thr | Exon 3 | NR | probably damaging (1.000) | Damaging (0.000) | Martín-Hernández (2014) |
| 104 | rs72554339 | c.264A>T | p.Lys88Asn | Exon 3 | pathogenic | probably damaging (1.000) | Damaging (0.000) | Reish (1993), Tuchman (1995a), Oppliger Leibundgut (1996a) |
| 105 | rs72554340 | c.268A>G | p.Ser90Gly | Exon 3 | pathogenic | probably damaging (1.000) | Damaging (0.000) | Takanashi (2002) |
| 106 | rs72554341 | c.269G>A | p.Ser90Asn | Exon 3 | pathogenic | probably damaging (1.000) | Damaging (0.000) | McCullough (2000) |
| 107 | rs72554342 | c.270T>G | p.Ser90Arg | Exon 3 | pathogenic | probably damaging (1.000) | Damaging (0.000) | Tuchman (1998), Shao (2017) |
| 108 | rs72554343 | c.271delA | p.Thr91Leufs*38 | Exon 3 | pathogenic | - | - | Genet (2000) |
| 109 | rs67418243 | c.274C>T | p.Arg92* | Exon 3 | pathogenic | - | - | Grompe (1991), Kim (2006), Storkanova (2013), Gilbert-Dussardier (1996) |
| 110 | rs67418243 | c.274C>G | p.Arg92Gly | Exon 3 | pathogenic | probably damaging (0.999) | Damaging (0.000) | Yamaguchi (2006) |
| 111 | rs66550389 | c.275G>A | p.Arg92Gln | Exon 3 | pathogenic | probably damaging (1.000) | Damaging (0.000) | Tuchman (1995a), Schultz (2000), Lu (2020), This study |
| 112 | rs66550389 | c.275G>T | p.Arg92Leu | Exon 3 | pathogenic | probably damaging (1.000) | Damaging (0.000) | Yamaguchi (2006) |
| 113 | rs66550389 | c.275G>C | p.Arg92Pro | Exon 3 | pathogenic | probably damaging (1.000) | Damaging (0.000) | Yamaguchi (2006) |
| 114 | rs72554344 | c.277A>G | p.Thr93Ala | Exon 3 | pathogenic | probably damaging (0.999) | Damaging (0.000) | Tuchman (1995a) |
| 115 |  | c.278C>T | p.Thr93Ile | Exon 3 | NR | probably damaging (1.000) | Damaging (0.000) | Martín-Hernández (2014) |
| 116 | rs72554345 | c.281G>C | p.Arg94Thr | Exon 3 | pathogenic | probably damaging (1.000) | Damaging (0.000) | Tuchman (1995a) |
| 117 | rs72554346 | c.284T>C | p.Leu95Ser | Exon 3 | pathogenic | probably damaging (0.960) | Damaging (0.001) | McCullough (2000) |
| 118 |  | c.286T>C | p.Ser96Pro | Exon 3 | uncertain significance | probably damaging (1.000) | Damaging (0.001) | Ali (2018) |
| 119 |  | c.287C>T | p.Ser96Phe | Exon 3 | NR | probably damaging (1.000) | Damaging (0.000) | Arranz (2007) |
| 120 | rs72554347 | c.292G>A | p.Glu98Lys | Exon 3 | uncertain significance | probably damaging (1.000) | Damaging (0.000) | Bisanzi (2002) |
| 121 |  | c.298delG | p.Gly100Alafs*21 | Exon 3 | NR | - | - | Gobin-Limballe (2021) |
| 122 |  | c.298G>T | p.Gly100Cys | Exon 3 | pathogenic | probably damaging (0.988) | Damaging (0.003) | Gobin-Limballe (2021) |
| 123 |  | c.298G>C | p.Gly100Asp | Exon 3 | NR | probably damaging (1.000) | Damaging (0.002) | Kim (2006) |
| 124 | rs68058881 | c.298+1G>A | - | Intron 3 | pathogenic | - | - | Garcia-Perez (1995b) |
| 125 | rs68058881 | c.298+1G>T | - | Intron 3 | pathogenic | - | - | Yamaguchi (2006) |
| 126 |  | c.298+1G>C | - | Intron 3 | NR | - | - | Martín-Hernández (2014) |
| 127 |  | c.298+2T>G | - | Intron 3 | pathogenic | - | - | Lu (2020) |
| 128 | rs72554348 | c.298+5G>C | - | Intron 3 | benign/Likely benign | - | - | Yamaguchi (2006), Lu (2020) |
| 129 | rs111060773 | c.298+1_298+5del | - | Intron 3 | pathogenic | - | - | Tuchman (1997) |
| 130 |  | c.299-8T>A | - | Intron 3 | benign | - | - | Caldovic (2015) |
| 131 |  | c.299-7A>G | - | Intron 3 | NR | - | - | Caldovic (2015) |
| 132 | rs72554349 | c.299G>A | p.Gly100Asp | Exon 4 | pathogenic | probably damaging (1.000) | Damaging (0.002) | Ali (2018), Oppliger Leibundgut (1997) |
| 133 |  | c.304G>C | p.Ala102Pro | Exon 4 | NR | probably damaging (0.987) | Damaging (0.014) | Storkanova (2013, Kido (2021) |
| 134 | rs72554350 | c.305C>A | p.Ala102Glu | Exon 4 | pathogenic | probably damaging (0.981) | Tolerated (0.089) | Tuchman (1995a) |
| 135 |  | c.313G>T | p.Gly105* | Exon 4 | NR | - | - | This study |
| 136 |  | c.314G>A | p.Gly105Glu | Exon 4 | NR | probably damaging (1.000) | Damaging (0.001) | Cavicchi (2014) |
| 137 | rs72554351 | c.314G>T | p.Gly105Val | Exon 4 | pathogenic | probably damaging (1.000) | Damaging (0.000) | Yamaguchi (2006) |
| 138 | rs72554352 | c.316G>A | p.Gly106Arg | Exon 4 | pathogenic | probably damaging (1.000) | Damaging (0.001) | McCullough (2000) |
| 139 | rs67651903 | c.317G>A | p.Gly106Glu | Exon 4 | pathogenic | probably damaging (1.000) | Damaging (0.001) | Takanashi (2002) |
| 140 | rs67651903 | c.317G>T | p.Gly106Val | Exon 4 | pathogenic | probably damaging (1.000) | Damaging (0.001) | Yamaguchi (2006, Lu (2020) |
| 141 |  | c.325T>C | p.Cys109Arg | Exon 4 | NR | benign (0.249) | Damaging (0.034) | Kido (2021) |
| 142 |  | c.327T>A | p.Cys109* | Exon 4 | pathogenic | - | - | Gobin-Limballe (2021) |
| 143 |  | c.327T>C | p.Cys109Arg | Exon 4 | NR | benign (0.249) | Damaging (0.034) | Caldovic (2015) |
| 144 | rs72554353 | c.330delT | p.Thr112Profs*9 | Exon 4 | pathogenic | - | - | Calvas (1998) |
| 145 | rs1800324 | c.332T>C | p.Leu111Pro | Exon 4 | pathogenic | probably damaging (1.000) | Damaging (0.008) | Grompe (1989) |
| 146 |  | c.341_342del | p.Gln114Argfs*8 | Exon 4 | NR | - | - | Azevedo (2006) |
| 147 | rs66539573 | c.350A>T | p.His117Leu | Exon 4 | pathogenic | probably damaging (0.996) | Damaging (0.006) | Tuchman (1995a) |
| 148 | rs66539573 | c.350A>G | p.His117Arg | Exon 4 | pathogenic | probably damaging (0.993) | Damaging (0.010) | Matsuda (1997) |
| 149 |  | c.350A>C | p.His117Pro | Exon 4 | NR | probably damaging (1.000) | Damaging (0.010) | Kido (2021) |
| 150 | rs72554354 | c.359_360del | p.Val120Glufs*2 | Exon 4 | pathogenic | - | - | Yamaguchi (2006) |
| 151 | rs72554355 | c.364_365insTT | p.Glu122Valfs*66 | Exon 4 | likely pathogenic | - | - | Yamaguchi (2006) |
| 152 | rs1131691517 | c.365A>G | p.Glu122Gly | Exon 4 | likely pathogenic | probably damaging (1.000) | Damaging (0.000) | Arranz (2007) |
| 153 |  | c.365A>T | p.Glu122Val | Exon 4 | NR | probably damaging (0.999) | Damaging (0.000) | Nguyen (2020) |
| 154 | rs72554356 | c.374C>T | p.Thr125Met | Exon 4 | uncertain significance | possibly damaging (0.557) | Tolerated (0.117) | Gilbert-Dussardier (1996) |
| 155 | rs72554357 | c.376delG | p.Asp126Thrfs*61 | Exon 4 | pathogenic | - | - | Yamaguchi (2006) |
| 156 | rs72554358 | c.377A>G | p.Asp126Gly | Exon 4 | pathogenic | probably damaging (1.000) | Damaging (0.000) | Matsuura (1994) |
| 157 | rs140046498 | c.385C>T | p.Arg129Cys | Exon 4 | uncertain significance | probably damaging (1.000) | Damaging (0.009) | Shchelochkov (2009) |
| 158 | rs66656800 | c.386G>A | p.Arg129His | Exon 4 | pathogenic | probably damaging (1.000) | Tolerated (0.072) | Matsuura (1994), Garcia-Perez (1995a), Genet (2000), Storkanova (2013), Lee (2014), Rivera-Barahona (2015), Shao (2017), Lee (2020), Kido (2021), This study |
| 159 | rs66656800 | c.386G>C | p.Arg129Pro | Exon 4 | pathogenic | probably damaging (1.000) | Tolerated (0.064) | Yamaguchi (2006) |
| 160 | rs66656800 | c.386G>T | p.Arg129Leu | Exon 4 | pathogenic | probably damaging (0.975) | Tolerated (0.163) | Strautnieks (1993), Lu (2020) |
| 161 | rs66737144 | c.386+1G>A | - | Intron 4 | pathogenic | - | - | Yamaguchi (2006), Martín-Hernández (2014) |
| 162 | rs66737144 | c.386+1G>T | - | Intron 4 | pathogenic | - | - | Yamaguchi (2006) |
| 163 |  | c.386+1G>C | - | Intron 4 | NR | - | - | Ogino (2007) |
| 164 | rs72554359 | c.386+2T>C | - | Intron 4 | pathogenic | - | - | Yamaguchi (2006), Martín-Hernández (2014) |
| 165 |  | c.386+4delT | - | Intron 4 | NR | - | - | Mohamed (2015) |
| 166 |  | c.386+5G>A | - | Intron 4 | NR | - | - | Arranz (2007) |
| 167 | rs66556380 | c.387-2A>C | - | Intron 4 | pathogenic | - | - | McCullough (2000) |
| 168 | rs66556380 | c.387-2A>G | - | Intron 4 | pathogenic | - | - | McCullough (2000) |
| 169 | rs66556380 | c.387-2A>T | - | Intron 4 | pathogenic | - | - | Carstens (1991) |
| 170 | rs72556251 | c.390_392dup | p.Leu131dup | Exon 5 | pathogenic | - | - | Tuchman (2002) |
| 171 |  | c.391_397dup | p.Ser133Ilefs*3 | Exon 5 | pathogenic | - | - | Arranz (2007) |
| 172 | rs72556252 | c.392T>C | p.Leu131Ser | Exon 5 | pathogenic | probably damaging (1.000) | Damaging (0.001) | Yamaguchi (2006), Kido (2021) |
| 173 | rs72556253 | c.394T>C | p.Ser132Pro | Exon 5 | pathogenic | probably damaging (0.999) | Damaging (0.002) | Bisanzi (2002) |
| 174 | rs72556254 | c.395C>T | p.Ser132Phe | Exon 5 | pathogenic | probably damaging (1.000) | Damaging (0.000) | Gyato (2004) |
| 175 | rs72556255 | c.403delG | p.Ala135Glnfs*52 | Exon 5 | pathogenic | - | - | Tuchman (1992) |
| 176 | rs72556256 | c.404C>A | p.Ala135Glu | Exon 5 | pathogenic | benign (0.024) | Damaging (0.005) | Yamaguchi (2006) |
| 177 | rs72556257 | c.407A>T | p.Asp136Val | Exon 5 | pathogenic | probably damaging (0.993) | Damaging (0.013) | Yamaguchi (2006) |
| 178 | rs72556258 | c.409G>A | p.Ala137Thr | Exon 5 | pathogenic | possibly damaging (0.501) | Damaging (0.025) | Yamaguchi (2006) |
| 179 |  | c.409G>C | p.Ala137Pro | Exon 5 | NR | probably damaging (0.971) | Damaging (0.034) | Azevedo (2006) |
| 180 | rs72556259 | c.416T>C | p.Leu139Ser | Exon 5 | pathogenic | probably damaging (1.000) | Damaging (0.001) | Tuchman (1997) |
| 181 | rs72556260 | c.418G>C | p.Ala140Pro | Exon 5 | likely pathogenic | probably damaging (1.000) | Damaging (0.000) | Yamaguchi (2006), Kim (2006), Choi (2015) |
| 182 |  | c.419C>A | p.Ala140Asp | Exon 5 | NR | probably damaging (1.000) | Damaging (0.000) | Shchelochkov (2009) |
| 183 | rs67960011 | c.421C>T | p.Arg141* | Exon 5 | pathogenic | - | - | Hata (1989), Matsuura (1993), Ogino (2007), Shao (2017) |
| 184 | rs67960011 | c.421C>G | p.Arg141Gly | Exon 5 | pathogenic | probably damaging (1.000) | Damaging (0.000) | Yamaguchi (2006), Lu (2020) |
| 185 | rs68026851 | c.422G>A | p.Arg141Gln | Exon 5 | pathogenic | probably damaging (1.000) | Damaging (0.000) | Maddalena (1988), Tuchman (1995a), Yoo (1996), Bisanzi (2002), Kim (2006), Choi (2015), Ali (2018), Lu (2020), Kido (2021), This study |
| 186 | rs68026851 | c.422G>C | p.Arg141Pro | Exon 5 | likely pathogenic | probably damaging (1.000) | Damaging (0.000) | Tuchman (1997) |
| 187 | rs72556261 | c.425T>A | p.Val142Glu | Exon 5 | pathogenic | probably damaging (1.000) | Damaging (0.000) | Tuchman (2002) |
| 188 |  | c.429T>A | p.Tyr143* | Exon 5 | pathogenic | - | - | Mukhtar (2013) |
| 189 | rs72556262 | c.430A>T | p.Lys144* | Exon 5 | pathogenic | - | - | Tuchman (1995b) |
| 190 | rs72556263 | c.437C>G | p.Ser146* | Exon 5 | pathogenic | - | - | Genet (2000) |
| 191 | rs67016166 | c.443T>C | p.Leu148Ser | Exon 5 | uncertain significance | probably damaging (1.000) | Damaging (0.001) | Yamaguchi (2006) |
| 192 | rs67016166 | c.443T>G | p.Leu148Trp | Exon 5 | pathogenic | probably damaging (1.000) | Damaging (0.000) | McCullough (2000) |
| 193 | rs66741318 | c.444G>C | p.Leu148Phe | Exon 5 | pathogenic | probably damaging (0.999) | Damaging (0.003) | Komaki (1997) |
| 194 | rs66741318 | c.444G>T | p.Leu148Phe | Exon 5 | pathogenic | probably damaging (0.999) | Damaging (0.003) | Matsuura (1998) |
| 195 | rs72556264 | c.451delC | p.Leu151Trpfs*36 | Exon 5 | pathogenic | - | - | Tuchman (2002) |
| 196 | rs72556265 | c.452T>G | p.Leu151Arg | Exon 5 | pathogenic | probably damaging (1.000) | Damaging (0.000) | Yamaguchi (2006) |
| 197 | rs72556266 | c.455C>T | p.Ala152Val | Exon 5 | pathogenic | benign (0.406) | Damaging (0.016) | Kogo (1998) |
| 198 | rs72556267 | c.460G>T | p.Glu154* | Exon 5 | pathogenic | - | - | Grompe (1989) |
| 199 |  | c.461_471del | p.Glu154Alafs*18 | Exon 5 | NR | - | - | Storkanova (2013) |
| 200 | rs67890094 | c.463G>C | p.Ala155Pro | Exon 5 | pathogenic | probably damaging (0.995) | Damaging (0.007) | Yamaguchi (2006) |
| 201 | rs67890094 | c.463G>T | p.Ala155Ser | Exon 5 | NR | benign (0.148) | Tolerated (1.000) | Tuchman (2002) |
| 202 | rs72556268 | c.464C>A | p.Ala155Glu | Exon 5 | pathogenic | probably damaging (0.992) | Damaging (0.003) | Yamaguchi (2006) |
| 203 |  | c.472C>T | p.Pro158Ser | Exon 5 | NR | probably damaging (1.000) | Damaging (0.000) | Storkanova (2013) |
| 204 |  | c.473C>T | p.Pro158Leu | Exon 5 | pathogenic | probably damaging (1.000) | Damaging (0.000) | Gobin-Limballe (2021) |
| 205 | rs72556269 | c.476T>C | p.Ile159Thr | Exon 5 | pathogenic | probably damaging (0.999) | Damaging (0.001) | Garcia-Perez (1995b), Martín-Hernández (2014) |
| 206 |  | c.477T>G | p.Ile159Met | Exon 5 | NR | probably damaging (1.000) | Damaging (0.001) | Ben-Ari (2010) |
| 207 | rs67954347 | c.479T>A | p.Ile160Asn | Exon 5 | pathogenic | probably damaging (1.000) | Damaging (0.000) | Yamaguchi (2006) |
| 208 | rs67954347 | c.479T>C | p.Ile160Thr | Exon 5 | pathogenic | probably damaging (0.991) | Damaging (0.001) | Yamaguchi (2006) |
| 209 | rs67954347 | c.479T>G | p.Ile160Ser | Exon 5 | pathogenic | probably damaging (0.999) | Damaging (0.000) | Climent (2002) |
| 210 | rs72556270 | c.481A>G | p.Asn161Asp | Exon 5 | pathogenic | probably damaging (1.000) | Damaging (0.003) | Genet (2000), Martín-Hernández (2014) |
| 211 | rs72556271 | c.482A>G | p.Asn161Ser | Exon 5 | pathogenic | probably damaging (1.000) | Damaging (0.018) | Tuchman (1995a, 1995b), Bisanzi (2002), Kim (2006), Shao (2017), Chongsrisawat (2018), Lu (2020) |
| 212 |  | c.483T>A | p.Asn161Lys | Exon 5 | NR | probably damaging (1.000) | Damaging (0.001) | Takanashi (2002) |
| 213 | rs66626662 | c.484G>A | p.Gly162Arg | Exon 5 | pathogenic | probably damaging (1.000) | Damaging (0.002) | Feldmann (1992), Tuchman (1995a) |
| 214 | rs66626662 | c.484G>C | p.Gly162Arg | Exon 5 | pathogenic | probably damaging (1.000) | Damaging (0.002) | Yamaguchi (2006) |
| 215 | rs72556272 | c.485G>A | p.Gly162Glu | Exon 5 | pathogenic | probably damaging (1.000) | Damaging (0.002) | Yamaguchi (2006) |
| 216 |  | c.488T>G | p.Leu163Arg | Exon 5 | pathogenic | probably damaging (1.000) | Damaging (0.000) | Gobin-Limballe (2021) |
| 217 | rs72556273 | c.490T>C | p.Ser164Pro | Exon 5 | pathogenic | probably damaging (1.000) | Tolerated (0.058) | Yamaguchi (2006) |
| 218 | rs72556274 | c.491C>G | p.Ser164* | Exon 5 | pathogenic | - | - | Hoshide (1993), Matsuda (1997), Storkanova (2013) |
| 219 | rs72556275 | c.493G>T | p.Asp165Tyr | Exon 5 | pathogenic | probably damaging (1.000) | Damaging (0.001) | Genet (2000) |
| 220 | rs66564822 | c.501C>A | p.Tyr167* | Exon 5 | pathogenic | - | - | Garcia-Perez (1995b) |
| 221 | rs66564822 | c.501C>G | p.Tyr167* | Exon 5 | pathogenic | - | - | Shimadzu (1998) |
| 222 | rs66867430 | c.503A>C | p.His168Pro | Exon 5 | Pathogenic | probably damaging (1.000) | Damaging (0.000) | Yamaguchi (2006) |
| 223 | rs66867430 | c.503A>G | p.His168Arg | Exon 5 | pathogenic | probably damaging (1.000) | Damaging (0.000) | Vella (1996) |
| 224 | rs72556276 | c.504T>A | p.His168Gln | Exon 5 | pathogenic | probably damaging (1.000) | Damaging (0.000) | Tuchman (1997) |
| 225 |  | c.505C>T | p.Pro169Ser | Exon 5 | likely pathogenic | probably damaging (1.000) | Damaging (0.000) | Genet (2000), Kido (2021) |
| 226 | rs72556277 | c.505C>G | p.Pro169Ala | Exon 5 | likely pathogenic | probably damaging (1.000) | Damaging (0.000) | Tuchman (2002) |
| 227 |  | c.506C>A | p.Pro169His | Exon 5 | NR | probably damaging (1.000) | Damaging (0.000) | Caldovic (2015) |
| 228 | rs72556278 | c.506C>T | p.Pro169Leu | Exon 5 | pathogenic | probably damaging (1.000) | Damaging (0.000) | Genet (2000) |
| 229 |  | c.511C>G | p.Gln171Glu | Exon 5 | NR | probably damaging (1.000) | Damaging (0.000) | Shchelochkov (2009) |
| 230 |  | c.513G>T | p.Gln171His | Exon 5 | NR | probably damaging (1.000) | Damaging (0.000) | Ali (2018) |
| 231 | rs72556279 | c.514A>T | p.Ile172Phe | Exon 5 | pathogenic | probably damaging (0.999) | Damaging (0.016) | Climent (1999) |
| 232 |  | c.515T>A | p.Ile172Asn | Exon 5 | NR | probably damaging (1.000) | Damaging (0.012) | Ogino (2007, Kido (2021) |
| 233 |  | c.516_525del | p.Leu173Thrfs*11 | Exon 5 | NR | - | - | Arranz (2007) |
| 234 | rs72556280 | c.516C>G | p.Ile172Met | Exon 5 | pathogenic | probably damaging (0.998) | Tolerated (0.051) | Matsuura (1994), Shao (2017) |
| 235 | rs72556281 | c.520G>C | p.Ala174Pro | Exon 5 | pathogenic | probably damaging (1.000) | Damaging (0.002) | Tsai (1993), Tuchman (1995a) |
| 236 |  | c.523_536del | p.Asp175Profs*5 | Exon 5 | NR | - | - | Kido (2021) |
| 237 | rs68033093 | c.524A>G | p.Asp175Gly | Exon 5 | pathogenic | probably damaging (1.000) | Damaging (0.003) | Genet (2000) |
| 238 | rs68033093 | c.524A>T | p.Asp175Val | Exon 5 | pathogenic | probably damaging (1.000) | Damaging (0.000) | Tuchman (1997) |
| 239 | rs72556282 | c.526T>C | p.Tyr176His | Exon 5 | Pathogenic | probably damaging (1.000) | Damaging (0.001) | Tuchman (2002) |
| 240 | rs72556283 | c.527A>G | p.Tyr176Cys | Exon 5 | pathogenic | probably damaging (1.000) | Damaging (0.002) | Oppliger Leibundgut (1996a) |
| 241 |  | c.527A>C | p.Tyr176Ser | Exon 5 | NR | probably damaging (1.000) | Damaging (0.002) | Azevedo (2006) |
| 242 |  | c.530_533dup | p.Leu179Hisfs*7 | Exon 5 | NR | - | - | Gilbert-Dussardier (1996) |
| 243 |  | c.530T>G | p.Leu177Arg | Exon 5 | uncertain significance | probably damaging (0.999) | Damaging (0.002) | Kido (2021) |
| 244 | rs72556285 | c.532_537del | p.Thr178_Leu179del | Exon 5 | pathogenic | - |  | Shimadzu (1998) |
| 245 | rs72556284 | c.533C>T | p.Thr178Met | Exon 5 | pathogenic | probably damaging (1.000) | Damaging (0.000) | Oppliger Leibundgut (1995), Bisanzi (2002), Choi (2015), Kido (2021) |
| 246 |  | c.535C>T | p.Leu179Phe | Exon 5 | likely pathogenic | probably damaging (0.998) | Damaging (0.007) | Arranz (2007), Fantur (2013), Kido (2021) |
| 247 | rs72556286 | c.536T>C | p.Leu179Pro | Exon 5 | pathogenic | probably damaging (1.000) | Damaging (0.001) | Yamaguchi (2006) |
| 248 |  | c.538C>T | p.Gln180* | Exon 5 | NR | - | - | Caldovic (2015) |
| 249 |  | c.539_540AG>CC | p.Gln180Pro | Exon 5 | NR | probably damaging (1.000) | Tolerated (0.051) | Hübler (2001) |
| 250 | rs72556287 | c.540G>C | p.Gln180His | Exon 5 | pathogenic | probably damaging (1.000) | Tolerated (0.447) | Tuchman (1995a), Shimadzu (1998), Lu (2020), Zhou (2020) |
| 251 |  | c.540+1G>A | - | Intron 5 | NR | - | - | Laróvere (2018) |
| 252 | rs72556288 | c.540+1G>C | - | Intron 5 | pathogenic | - | - | Oppliger Leibundgut (1996b) |
| 253 | rs67367843 | c.540+2T>A | - | Intron 5 | pathogenic | - | - | Yamaguchi (2006) |
| 254 |  | c.540+2T>G | - | Intron 5 | NR | - | - | Shchelochkov (2009) |
| 255 | rs67367843 | c.540+2T>C | - | Intron 5 | pathogenic | - | - | Matsuura (1995) |
| 256 |  | c.540+5G>A | - | Intron 5 | NR | - | - | Tuchman (1997) |
| 257 |  | c.540+265G>A | - | Intron 5 | pathogenic | - | - | Ogino (2007), Lu (2020), Kumar (2021) |
| 258 | rs72556289 | c.541-2A>G | - | Intron 5 | pathogenic | - | - | Genet (2000) |
| 259 | rs72556290 | c.542A>G | p.Glu181Gly | Exon 6 | pathogenic | probably damaging (0.969) | Damaging (0.032) | Tuchman (1998) |
| 260 | rs72556291 | c.545A>T | p.His182Leu | Exon 6 | pathogenic | probably damaging (1.000) | Tolerated (0.411) | Tuchman (1995a) |
| 261 | rs72556292 | c.547T>G | p.Tyr183Asp | Exon 6 | Pathogenic | probably damaging (1.000) | Damaging (0.002) | Oppliger Leibundgut (1997) |
| 262 | rs72556293 | c.548A>G | p.Tyr183Cys | Exon 6 | pathogenic | probably damaging (1.000) | Damaging (0.002) | Tuchman (1995a), Storkanova (2013), Lee (2014), Choi (2015) |
| 263 |  | c.552insGAAC | p.Ser185Efs*41 | Exon 6 | NR | - | - | Lu (2020) |
| 264 |  | c.557T>C | p.Leu186Pro | Exon 6 | NR | probably damaging (1.000) | Damaging (0.016) | Azevedo (2006) |
| 265 |  | c.561delA | p.Gly188Valfs*18 | Exon 6 | NR | - | - | Caldovic (2015) |
| 266 | rs796052016 | c.562_563del | p.Gly188Serfs*36 | Exon 6 | pathogenic | - | - | Shchelochkov (2009) |
| 267 | rs72556294 | c.562G>C | p.Gly188Arg | Exon 6 | pathogenic | probably damaging (1.000) | Damaging (0.001) | Gilbert-Dussardier (1996), Lu (2020) |
| 268 | rs72556295 | c.563G>T | p.Gly188Val | Exon 6 | pathogenic | probably damaging (1.000) | Damaging (0.000) | Climent (1999) |
| 269 |  | c.563G>C | p.Gly188Ala | Exon 6 | NR | probably damaging (1.000) | Damaging (0.001) | Shchelochkov (2009) |
| 270 |  | c.568delA | p.Thr190Profs*16 | Exon 6 | NR | - | - | Shchelochkov (2009) |
| 271 |  | c.568dupA | p.Thr190Asnfs*35 | Exon 6 | pathogenic | - | - | Gobin-Limballe (2021) |
| 272 |  | c.571delC | p.Leu191Serfs*15 | Exon 6 | NR | - | - | Kim (2006) |
| 273 | rs72556296 | c.571C>T | p.Leu191Phe | Exon 6 | pathogenic | benign (0.074) | Tolerated (0.081) | Climent (2002) |
| 274 | rs72556297 | c.572T>G | p.Leu191Arg | Exon 6 | uncertain significance | probably damaging (0.992) | Damaging (0.002) | Yamaguchi (2006) |
| 275 | rs72556298 | c.576C>G | p.Ser192Arg | Exon 6 | pathogenic | probably damaging (0.997) | Damaging (0.001) | Matsuura (1993) |
| 276 | rs67284661 | c.577T>C | p.Trp193Arg | Exon 6 | likely pathogenic | probably damaging (1.000) | Damaging (0.001) | Yamaguchi (2006) |
| 277 | rs67284661 | c.577T>G | p.Trp193Gly | Exon 6 | pathogenic | probably damaging (1.000) | Damaging (0.003) | Yamaguchi (2006) |
| 278 | rs72556299 | c.578G>A | p.Trp193* | Exon 6 | pathogenic | - | - | Shimadzu (1998), Ogino (2007) |
| 279 |  | c.579G>A | p.Trp193* | Exon 6 | NR | - | - | Lu (2020) |
| 280 |  | c.579G>C | p.Trp193Cys | Exon 6 | NR | probably damaging (1.000) | Damaging (0.009) | Shchelochkov (2009) |
| 281 |  | c.581T>C | p.Ile194Thr | Exon 6 | NR | probably damaging (0.999) | Damaging (0.001) | Caldovic (2015) |
| 282 | rs67294955 | c.583G>A | p.Gly195Arg | Exon 6 | pathogenic | probably damaging (1.000) | Damaging (0.006) | Tuchman (1994, 1995), Choi (2015), Ali (2018), Lu (2020), Zhou (2020), Kido (2021) |
| 283 |  | c.583G>C | p.Gly195Arg | Exon 6 | NR | probably damaging (1.000) | Damaging (0.006) | Kim (2006), Storkanova (2013), Choi (2015) |
| 284 |  | c.584G>C | p.Gly195Ala | Exon 6 | pathogenic | probably damaging (1.000) | Damaging (0.002) | Gobin-Limballe (2021) |
| 285 | rs67294956 | c.586delG | p.Asp196Metfs*10 | Exon 6 | pathogenic | - | - | Climent (2002) |
| 286 | rs66642398 | c.586G>A | p.Asp196Asn | Exon 6 | pathogenic | probably damaging (1.000) | Damaging (0.000) | Yamaguchi (2006), Shao (2017) |
| 287 | rs66642398 | c.586G>T | p.Asp196Tyr | Exon 6 | pathogenic | probably damaging (1.000) | Damaging (0.000) | Tuchman (1998), Azevedo (2006) |
| 288 |  | c.586G>C | p.Asp196His | Exon 6 | NR | probably damaging (1.000) | Damaging (0.000) | Lin (2010) |
| 289 | rs72556300 | c.587A>T | p.Asp196Val | Exon 6 | pathogenic | probably damaging (1.000) | Damaging (0.000) | Matsuura (1993) |
| 290 | rs72556301 | c.589G>A | p.Gly197Arg | Exon 6 | pathogenic | probably damaging (1.000) | Damaging (0.002) | Climent (1999) |
| 291 |  | c.589G>T | p.Gly197Trp | Exon 6 | NR | probably damaging (1.000) | Damaging (0.000) | Shchelochkov (2009) |
| 292 | rs72556302 | c.590G>A | p.Gly197Glu | Exon 6 | pathogenic | probably damaging (1.000) | Damaging (0.003) | Tuchman (1998) |
| 293 | rs72558403 | c.593A>T | p.Asn198Ile | Exon 6 | pathogenic | probably damaging (1.000) | Damaging (0.000) | Yamaguchi (2006) |
| 294 | rs72558404 | c.594C>A | p.Asn198Lys | Exon 6 | Pathogenic | probably damaging (1.000) | Damaging (0.000) | Popowska (1999) |
| 295 | rs72558405 | c.595A>G | p.Asn199Asp | Exon 6 | pathogenic | probably damaging (1.000) | Damaging (0.000) | Yamaguchi (2006), Ali (2018), Liu (2021) |
| 296 |  | c.595A>C | p.Asn199His | Exon 6 | NR | probably damaging (1.000) | Damaging (0.000) | Ali (2018) |
| 297 | rs72558406 | c.596A>G | p.Asn199Ser | Exon 6 | pathogenic/likely pathogenic | probably damaging (1.000) | Damaging (0.000) | Tuchman (2002) |
| 298 |  | c.597_598del | p.Ile200Profs*24 | Exon 6 | NR | - | - | Tuchman (1994) |
| 299 |  | c.601C>A | p.Leu201Met | Exon 6 | NR | probably damaging (1.000) | Damaging (0.003) | Shao (2017) |
| 300 | rs72558407 | c.602T>C | p.Leu201Pro | Exon 6 | pathogenic | probably damaging (1.000) | Damaging (0.000) | Shimadzu (1998) |
| 301 | rs72558408 | c.604C>T | p.His202Tyr | Exon 6 | pathogenic | probably damaging (0.998) | Tolerated (0.143) | Tuchman (1997), Lu (2020), Kido (2021) |
| 302 | rs72558409 | c.605A>C | p.His202Pro | Exon 6 | pathogenic | probably damaging (1.000) | Damaging (0.028) | Staudt (1998) |
| 303 |  | c.605A>T | p.His202Leu | Exon 6 | NR | probably damaging (0.980) | Tolerated (0.067) | Martín-Hernández (2014) |
| 304 |  | c.607T>C | p.Ser203Pro | Exon 6 | NR | probably damaging (1.000) | Damaging (0.003) | Bernal (2021) |
| 305 |  | c.608C>T | p.Ser203Phe | Exon 6 | Conflicting interpretations of pathogenicity | probably damaging (1.000) | Damaging (0.001) | Gobin-Limballe (2021) |
| 306 | rs72558410 | c.608C>G | p.Ser203Cys | Exon 6 | pathogenic | probably damaging (1.000) | Damaging (0.001) | Tuchman (1995a) |
| 307 | rs72558411 | c.613A>G | p.Met205Val | Exon 6 | likely pathogenic | probably damaging (0.997) | Tolerated (0.515) | Genet (2000) |
| 308 |  | c.614T>C | p.Met205Thr | Exon 6 | NR | probably damaging (0.997) | Tolerated (0.161) | Kim (2006), Kido (2021) |
| 309 | rs72558412 | c.617T>G | p.Met206Arg | Exon 6 | pathogenic | possibly damaging (0.722) | Damaging (0.001) | Tuchman (1997), Kim (2006), Kido (2021) |
| 310 | rs72558413 | c.618G>C | p.Met206Ile | Exon 6 | pathogenic | benign (0.013) | Tolerated (1.000) | Climent (2002) |
| 311 | rs72558414 | c.620G>A | p.Ser207Asn | Exon 6 | pathogenic | possibly damaging (0.565) | Damaging (0.002) | Yamaguchi (2006), Storkanova (2013) |
| 312 | rs72558415 | c.621C>A | p.Ser207Arg | Exon 6 | pathogenic | probably damaging (0.999) | Damaging (0.002) | Shimadzu (1998) |
| 313 | rs72558416 | c.622G>A | p.Ala208Thr | Exon 6 | pathogenic | probably damaging (0.999) | Damaging (0.011) | Diggelen (2008), Schultz (2000), Bisanzi (2002), Cavicchi (2014), Lu (2020) |
| 314 |  | c.626C>A | p.Ala209Glu | Exon 6 | pathogenic | probably damaging (0.999) | Tolerated (0.064) | Bailly (2015) |
| 315 | rs72558417 | c.626C>T | p.Ala209Val | Exon 6 | pathogenic | probably damaging (0.997) | Tolerated (0.796) | Garcia-Perez (1995b), Gilbert-Dussardier (1996), Takanashi (2002), Lu (2020), Zhou (2020), Kido (2021) |
| 316 | rs72558418 | c.628A>C | p.Lys210Gln | Exon 6 | pathogenic | possibly damaging (0.897) | Damaging (0.017) | Valik (2004), Storkanova (2013) |
| 317 |  | c.628A>G | p.Lys210Glu | Exon 6 | NR | probably damaging (0.993) | Damaging (0.002) | Storkanova (2013) |
| 318 |  | c.630delA | p.Lys210Asnfs*20 | Exon 6 | NR | - | - | Martín-Hernández (2014) |
| 319 |  | c.630A>C | p.Lys210Asn | Exon 6 | NR | probably damaging (1.000) | Damaging (0.001) | Azevedo (2006) |
| 320 |  | c.635G>T | p.Gly212Val | Exon 6 | pathogenic | probably damaging (1.000) | Damaging (0.001) | Gobin-Limballe (2021) |
| 321 |  | c.638T>A | p.Met213Lys | Exon 6 | NR | probably damaging (0.973) | Damaging (0.005) | Oppliger Leibundgut (1997) |
| 322 |  | c.638T>G | p.Met213Arg | Exon 6 | NR | probably damaging (0.996) | Damaging (0.003) | Caldovic (2015) |
| 323 |  | c.638T>C | p.Met213Thr | Exon 6 | NR | probably damaging (0.992) | Damaging (0.020) | Caldovic (2015), Ali (2018) |
| 324 | rs72558420 | c.640C>T | p.His214Tyr | Exon 6 | pathogenic | possibly damaging (0.938) | Damaging (0.028) | Yoo (1996), Kim (2006), Choi (2015) |
| 325 | rs72558421 | c.643C>T | p.Leu215Phe | Exon 6 | pathogenic | probably damaging (0.968) | Tolerated (0.057) | Ueta (2001), Kido (2021) |
| 326 | rs72558422 | c.645dupT | p.Gln216Serfs*9 | Exon 6 | pathogenic | - | - | Tuchman (1994b) |
| 327 | rs72558423 | c.646C>G | p.Gln216Glu | Exon 6 | pathogenic | possibly damaging (0.835) | Tolerated (0.340) | Grompe (1989) |
| 328 | rs72558424 | c.650C>A | p.Ala217Glu | Exon 6 | pathogenic | possibly damaging (0.507) | Damaging (0.001) | Yamaguchi (2006) |
| 329 |  | c.652G>A | p.Ala218Thr | Exon 6 | NR | probably damaging (1.000) | Damaging (0.001) | Hwu (2003a), This study |
| 330 |  | c.652G>C | p.Ala218Pro | Exon 6 | uncertain significance | probably damaging (1.000) | Damaging (0.002) | Gobin-Limballe (2021) |
| 331 |  | c.653C>T | p.Ala218Val | Exon 6 | NR | probably damaging (1.000) | Damaging (0.017) | Shchelochkov (2009) |
| 332 |  | c.658C>A | p.Pro220Thr | Exon 6 | NR | probably damaging (1.000) | Damaging (0.000) | Arranz (2007) |
| 333 | rs72558425 | c.658C>G | p.Pro220Ala | Exon 6 | pathogenic | probably damaging (1.000) | Damaging (0.000) | Oppliger Leibundgut (1996a) |
| 334 | rs72558426 | c.659C>T | p.Pro220Leu | Exon 6 | pathogenic | probably damaging (1.000) | Damaging (0.000) | Yamaguchi (2006) |
| 335 | rs281865552 | c.663G>A | p.Lys221= | Exon 6 | pathogenic | - | - | Shimadzu (1998) |
| 336 |  | c.663G>T | p.Lys221Asn | Exon 6 | NR | probably damaging (0.996) | Damaging (0.022) | Kim (2006) |
| 337 | rs281865552 | c.663G>C | p.Lys221Asn | Exon 6 | pathogenic | probably damaging (0.996) | Damaging (0.022) | Yamaguchi (2006) |
| 338 |  | c.663+1delG | - | Intron 6 | NR | - | - | Shchelochkov (2009) |
| 339 | rs68170503 | c.663+1G>A | - | Intron 6 | pathogenic | - | - | Tuchman (1997) |
| 340 | rs68170503 | c.663+1G>T | - | Intron 6 | pathogenic | - | - | Oppliger Leibundgut (1996b) |
| 341 | rs72558427 | c.663+2T>C | - | Intron 6 | pathogenic | - | - | Tuchman (1997) |
| 342 |  | c.663+2dupT | - | Intron 6 | NR | - | - | Tuchman (1995b) |
| 343 | rs67839039 | c.664-1delG | - | Intron 6 | pathogenic | - | - | Tuchman (1997) |
| 344 | rs67839036 | c.664-1G>A | - | Intron 6 | pathogenic | - | - | Tuchman (2002), Climent (2002), Lu (2020) |
| 345 |  | c.664_667delinsAC | p.Gly222Thrfs*2 | Exon 7 | NR | - | - | Lee (2014), Choi (2015) |
| 346 |  | c.670G>T | p.Glu224* | Exon 7 | NR | - | - | Shchelochkov (2009) |
| 347 | rs72558428 | c.673C>A | p.Pro225Thr | Exon 7 | pathogenic | possibly damaging (0.790) | Tolerated (0.419) | Tuchman (1995a) |
| 348 | rs67120076 | c.674C>G | p.Pro225Arg | Exon 7 | pathogenic | probably damaging (1.000) | Tolerated (0.359) | Garcia-Perez (1995b) |
| 349 | rs67120076 | c.674C>T | p.Pro225Leu | Exon 7 | pathogenic | probably damaging (1.000) | Tolerated (0.725) | Tuchman (1995a), Shao (2017) |
| 350 |  | c.674_675CG>AA | p.Pro225Gln | Exon 7 | NR | probably damaging (1.000) | Tolerated (0.296) | Kido (2021) |
| 351 |  | c.697delG | p.Leu232Leufs*14 | Exon 7 | NR | - | - | Laróvere (2018) |
| 352 | rs72558429 | c.698C>T | p.Ala233Val | Exon 7 | pathogenic | probably damaging (0.965) | Tolerated (0.107) | Yamaguchi (2006) |
| 353 | rs72558430 | c.700G>T | p.Glu234* | Exon 7 | pathogenic | - | - | Yamaguchi (2006) |
| 354 |  | c.703C>T | p.Gln235* | Exon 7 | NR | - | - | Lu (2020) |
| 355 |  | c.704A>C | p.Gln235Pro | Exon 7 | NR | possibly damaging (0.943) | Damaging (0.046) | Lu (2020) |
| 356 |  | c.710C>A | p.Ala237Asp | Exon 7 | NR | probably damaging (0.987) | Damaging (0.003) | Kim (2006) |
| 357 | rs67283833 | c.716A>T | p.Glu239Val | Exon 7 | pathogenic | possibly damaging (0.513) | Damaging (0.032) | Yamaguchi (2006) |
| 358 | rs67283833 | c.716A>G | p.Glu239Gly | Exon 7 | pathogenic | probably damaging (0.988) | Tolerated (0.102) | Yamaguchi (2006) |
| 359 | rs66851495 | c.717G>A | p.Glu239= | Exon 7 | uncertain significance | - | - | Tuchman (1997) |
| 360 | rs66851495 | c.717G>C | p.Glu239Asp | Exon 7 | likely pathogenic | possibly damaging (0.759) | Tolerated (0.084) | Yamaguchi (2006) |
| 361 | rs66500027 | c.717+1G>A | - | Intron 7 | pathogenic | - | - | Genet (2000), Nguyen (2020) |
| 362 | rs66500027 | c.717+1G>T | - | Intron 7 | pathogenic | - | - | Tuchman (2002), Storkanova (2013) |
| 363 | rs72558431 | c.717+2T>C | - | Intron 7 | pathogenic | - | - | Carstens (1991), Azevedo (2006) |
| 364 | rs72558432 | c.717+3A>G | - | Intron 7 | pathogenic | - | - | Carstens (1991), Choi (2015) |
| 365 | rs111060774 | c.717+8_717+23del | - | Intron 7 | pathogenic | - | - | Calvas (1998) |
| 366 | rs72558434 | c.718-2_731del | - | Intron 7 | pathogenic | - | - | Yamaguchi (2006) |
| 367 | rs72558433 | c.718-2A>G | - | Intron 7 | pathogenic | - | - | Popowska (1999) |
| 368 |  | c.718-1G>A | - | Intron 7 | NR | - | - | Lu (2020) |
| 369 | rs72558435 | c.725C>T | p.Thr242Ile | Exon 8 | pathogenic | probably damaging (0.999) | Damaging (0.036) | Tuchman (1997), Lu (2020) |
| 370 | rs72558438 | c.731_739del | p.Leu244_Thr247delinsPro | Exon 8 | pathogenic | - | - | Calvas (1998) |
| 371 | rs72558436 | c.731T>A | p.Leu244Gln | Exon 8 | pathogenic | probably damaging (0.995) | Damaging (0.002) | Calvas (1998) |
| 372 | rs72558437 | c.740C>A | p.Thr247Lys | Exon 8 | pathogenic | possibly damaging (0.921) | Damaging (0.016) | Tuchman (1995a) |
| 373 |  | c.740C>G | p.Thr247Arg | Exon 8 | pathogenic | probably damaging (0.984) | Damaging (0.001) | Gobin-Limballe (2021) |
| 374 |  | c.746A>G | p.Asp249Gly | Exon 8 | NR | possibly damaging (0.756) | Tolerated (0.076) | Kim (2006), Choi (2015) |
| 375 |  | c.749C>T | p.Pro250Leu | Exon 8 | NR | probably damaging (1.000) | Damaging (0.006) | Caldovic (2015) |
| 376 | rs67330615 | c.757G>A | p.Ala253Thr | Exon 8 | pathogenic | probably damaging (1.000) | Damaging (0.001) | Yamaguchi (2006) |
| 377 | rs67330615 | c.757G>C | p.Ala253Pro | Exon 8 | pathogenic | probably damaging (1.000) | Damaging (0.001) | Yamaguchi (2006) |
| 378 | rs72558439 | c.759delA | p.Ala254Argfs*7 | Exon 8 | Pathogenic | - | - | Yamaguchi (2006) |
| 379 |  | c.760A>T | p.Ala254* | Exon 8 | NR | - | - | Caldovic (2015) |
| 380 | rs72558440 | c.764A>C | p.His255Pro | Exon 8 | pathogenic | possibly damaging (0.511) | Damaging (0.011) | Tuchman (1998) |
| 381 |  | c.766G>T | p.Gly256* | Exon 8 | pathogenic | - | - | Gobin-Limballe (2021) |
| 382 |  | c.773_790del | p.Asn258_263del | Exon 8 | NR | - | - | Bijarnia-Mahay (2018) |
| 383 | rs72558441 | c.779T>C | p.Leu260Ser | Exon 8 | pathogenic | probably damaging (1.000) | Damaging (0.001) | Yamaguchi (2006), Lu (2020) |
| 384 |  | c.782T>C | p.Ile261Thr | Exon 8 | NR | probably damaging (0.980) | Damaging (0.002) | Li (2018) |
| 385 |  | c.784_792dup | p.Thr262_Thr264dup | Exon 8 | NR | - | - | Caldovic (2015) |
| 386 | rs67333670 | c.785C>A | p.Thr262Lys | Exon 8 | pathogenic | probably damaging (1.000) | Damaging (0.001) | Giorgi (2000) |
| 387 | rs67333670 | c.785C>T | p.Thr262Ile | Exon 8 | pathogenic | probably damaging (1.000) | Damaging (0.000) | Yamaguchi (2006), Shao (2017) |
| 388 | rs72558442 | c.787G>A | p.Asp263Asn | Exon 8 | pathogenic | probably damaging (1.000) | Damaging (0.000) | Tuchman (1997) |
| 389 | rs72558443 | c.788A>G | p.Asp263Gly | Exon 8 | pathogenic | probably damaging (1.000) | Damaging (0.000) | Tuchman (1998) |
| 390 | rs72558444 | c.790A>G | p.Thr264Ala | Exon 8 | pathogenic | probably damaging (0.998) | Damaging (0.002) | Matsuura (1993), Giorgi (2000), Kido (2021) |
| 391 | rs67156896 | c.791C>A | p.Thr264Asn | Exon 8 | pathogenic | probably damaging (0.959) | Damaging (0.001) | Hwu (2003b) |
| 392 | rs67156896 | c.791C>T | p.Thr264Ile | Exon 8 | uncertain significance | probably damaging (1.000) | Damaging (0.001) | Shimadzu (1998) |
| 393 | rs72558445 | c.793T>C | p.Trp265Arg | Exon 8 | pathogenic | probably damaging (1.000) | Damaging (0.032) | Yamaguchi (2006) |
| 394 |  | c.794G>A | p.Trp265* | Exon 8 | NR | - | - | Lu (2020) |
| 395 | rs72558446 | c.794G>T | p.Trp265Leu | Exon 8 | pathogenic | probably damaging (1.000) | Damaging (0.003) | Giorgi (2000) |
| 396 | rs72558447 | c.795G>A | p.Trp265* | Exon 8 | pathogenic | - | - | Yamaguchi (2006) |
| 397 |  | c.796_805del | p.Ile265_Gly268delinsAspfs*19 | Exon 8 | NR | - | - | Kim (2006), Choi (2015) |
| 398 |  | c.799A>C | p.Ser267Arg | Exon 8 | pathogenic | probably damaging (1.000) | Damaging (0.003) | Shimadzu (1998) |
| 399 |  | c.799_800insA | p.Ser267Lysfs*26 | Exon 8 | NR | - | - | Choi (2015) |
| 400 |  | c.802A>G | p.Met268Val | Exon 8 | NR | probably damaging (1.000) | Damaging (0.002) | Jamroz (2013) |
| 401 | rs72558449 | c.803T>C | p.Met268Thr | Exon 8 | pathogenic | probably damaging (1.000) | Damaging (0.000) | Matsuura (1993), Bisanzi (2002), Zhou (2020) |
| 402 |  | c.805G>A | p.Gly269Arg | Exon 8 | NR | probably damaging (1.000) | Damaging (0.000) | Shao (2017) |
| 403 | rs72558450 | c.806G>A | p.Gly269Glu | Exon 8 | likely pathogenic | probably damaging (1.000) | Damaging (0.000) | Zimmer (1995) |
| 404 | rs72558451 | c.808C>T | p.Gln270* | Exon 8 | pathogenic | - | - | McCullough (2000) |
| 405 | rs1800328 | c.809A>C | p.Gln270Pro | Exon 8 | pathogenic | benign (0.284) | Damaging (0.035) | Yamaguchi (2006) |
| 406 |  | c.813_814delAGinsC | p.Glu271Aspfs*28 | Exon 8 | NR | - | - | Khoo (1999), Ali (2018) |
| 407 | rs72558452 | c.817_819del | p.Glu273del | Exon 8 | Conflicting interpretations of pathogenicity | - | - | Ségues (1996), Schultz (2000), Martín-Hernández (2014) |
| 408 | rs72558453 | c.818delA | p.Glu273Glyfs*16 | Exon 8 | pathogenic | - | - | Yamaguchi (2006) |
| 409 |  | c.823A>T | p.Lys275* | Exon 8 | NR | - | - | Kido (2021) |
| 410 | rs72558454 | c.829C>T | p.Arg277Trp | Exon 8 | pathogenic | probably damaging (1.000) | Damaging (0.000) | Hata (1991), Matsuura (1993), Kim (2006), Storkanova (2013), Cavicchi (2014), Choi (2015), Shao (2017), Lee (2020), Lu (2020), Kido (2021) |
| 411 | rs66724222 | c.830G>A | p.Arg277Gln | Exon 8 | pathogenic | probably damaging (1.000) | Damaging (0.003) | Tuchman (1995a) |
| 412 | rs66724222 | c.830G>T | p.Arg277Leu | Exon 8 | pathogenic | probably damaging (1.000) | Damaging (0.001) | Tuchman (2002) |
| 413 |  | c.834_840del | p.Gln279Serfs*8 | Exon 8 | NR | - | - | Lee (2018), Kido (2021) |
| 414 | rs72558455 | c.835C>T | p.Gln279* | Exon 8 | pathogenic | - | - | Tuchman (2002) |
| 415 |  | c.842T>C | p.Phe281Ser | Exon 8 | NR | probably damaging (1.000) | Damaging (0.000) | Kim (2006) |
| 416 |  | c.850A>T | p.Tyr284Asn | Exon 8 | NR | probably damaging (1.000) | Damaging (0.000) | Chongsrisawat (2018) |
| 417 |  | c.852C>G | p.Tyr284* | Exon 8 | NR | - | - | Wu (2018) |
| 418 |  | c.853delC | p.Gln285Argfs*4 | Exon 8 | NR | - | - | Kim (2006) |
| 419 |  | c.853C>T | p.Gln285* | Exon 8 | NR | - | - | Storkanova (2013) |
| 420 |  | c.859A>C | p.Thr287Pro | Exon 8 | NR | probably damaging (1.000) | Damaging (0.001) | Caldovic (2015) |
| 421 |  | c.860C>T | p.Thr287Ile | Exon 8 | NR | probably damaging (0.998) | Damaging (0.001) | Lu (2020) |
| 422 |  | c.861_862insAC | p.Met288Thrfs*2 | Exon 8 | NR | - | - | Caldovic (2015) |
| 423 |  | c.867G>A | p.Lys289= | Exon 8 | NR | - | - | Storkanova (2013) |
| 424 | rs72558456 | c.867G>T | p.Lys289Asn | Exon 8 | pathogenic | possibly damaging (0.944) | Damaging (0.024) | Tuchman (2002) |
| 425 |  | c.867G>C | p.Lys289Asp | Exon 8 | NR | probably damaging (0.967) | Tolerated (0.376) | Caldovic (2015) |
| 426 | rs66512766 | c.867+1G>A | - | Intron 8 | pathogenic | - | - | Hoshide (1993), Tuchman (1998) |
| 427 | rs66512766 | c.867+1G>T | - | Intron 8 | pathogenic | - | - | Oppliger Leibundgut (1996b) |
| 428 |  | c.867+1G>C | - | Intron 8 | NR | - | - | Li (2018), Kido (2021) |
| 429 |  | c.867+1126A>G | - | Intron 8 | likely pathogenic | - | - | Engel (2008) |
| 430 |  | c.868-3T>C | - | Intron 8 | uncertain significance | - | - | Lee (2014) |
| 431 |  | c.868-1G>C | - | Intron 8 | NR | - | - | Lu (2020) |
| 432 | rs72558457 | c.876delA | p.Val293Leufs*30 | Exon 9 | pathogenic | - | - | Yamaguchi (2006) |
| 433 | rs72558458 | c.882delT | p.Ala295Profs*28 | Exon 9 | pathogenic | - | - | Reish (1993) |
| 434 |  | c.888delT | p.Asp297Thrfs*26 | Exon 9 | NR | - | - | Bernal (2021) |
| 435 |  | c.889G>T | p.Asp297Tyr | Exon 9 | NR | probably damaging (1.000) | Damaging (0.004) | Shchelochkov (2009) |
| 436 | rs72558459 | c.890_893del | p.Asp297Glyfs*25 | Exon 9 | pathogenic | - | - | Yamanouchi (2002) |
| 437 | rs72558460 | c.892_893del | p.Trp298Aspfs*15 | Exon 9 | pathogenic | - | - | Schimanski (1996) |
| 438 |  | c.892T>C | p.Trp298Arg | Exon 9 | NR | probably damaging (1.000) | Damaging (0.002) | Caldovic (2015) |
| 439 | rs72558461 | c.893G>C | p.Trp298Ser | Exon 9 | pathogenic | probably damaging (0.999) | Damaging (0.024) | Ensenauer (2005) |
| 440 |  | c.894G>A | p.Trp298* | Exon 9 | NR | - | - | Kido (2021) |
| 441 |  | c.902T>C | p.Leu301Ser | Exon 9 | NR | probably damaging (1.000) | Damaging (0.000) | Caldovic (2015) |
| 442 | rs72558462 | c.903A>T | p.Leu301Phe | Exon 9 | likely pathogenic | probably damaging (1.000) | Damaging (0.004) | Climent (2002) |
| 443 | rs72558463 | c.904C>T | p.His302Tyr | Exon 9 | pathogenic | probably damaging (1.000) | Damaging (0.000) | Oppliger Leibundgut (1996a), Lu (2020), Kido (2021) |
| 444 | rs67993095 | c.905A>G | p.His302Arg | Exon 9 | pathogenic | probably damaging (1.000) | Damaging (0.000) | Genet (2000) |
| 445 | rs67993095 | c.905A>T | p.His302Leu | Exon 9 | pathogenic | probably damaging (1.000) | Damaging (0.000) | Gilbert-Dussardier (1996) |
| 446 | rs67870245 | c.906delC | p.Cys303Alafs*20 | Exon 9 | pathogenic | - | - | Yamaguchi (2006) |
| 447 | rs67870244 | c.906C>G | p.His302Gln | Exon 9 | pathogenic | probably damaging (1.000) | Damaging (0.000) | Tuchman (1997) |
| 448 | rs67468335 | c.907T>C | p.Cys303Arg | Exon 9 | pathogenic | probably damaging (0.991) | Damaging (0.000) | Calvas (1998) |
| 449 | rs67468335 | c.907T>G | p.Cys303Gly | Exon 9 | pathogenic | probably damaging (1.000) | Damaging (0.000) | Tuchman (2002) |
| 450 | rs72558464 | c.908G>A | p.Cys303Tyr | Exon 9 | pathogenic | probably damaging (1.000) | Damaging (0.000) | Tuchman (1997) |
| 451 | rs72558465 | c.912G>T | p.Leu304Phe | Exon 9 | likely pathogenic | probably damaging (0.996) | Damaging (0.001) | Hoshide (1993) |
| 452 |  | c.912G>C | p.Leu304Phe | Exon 9 | NR | probably damaging (1.000) | Damaging (0.001) | Matsuura (1995), Kido (2021) |
| 453 |  | c.913C>T | p.Pro305Ser | Exon 9 | NR | probably damaging (1.000) | Damaging (0.000) | Lu (2020) |
| 454 | rs67501347 | c.914C>A | p.Pro305His | Exon 9 | pathogenic | probably damaging (1.000) | Damaging (0.000) | Climent (2002) |
| 455 |  | c.914C>T | p.Pro305Leu | Exon 9 | NR | probably damaging (1.000) | Damaging (0.000) | Bernal (2021) |
| 456 | rs67501347 | c.914C>G | p.Pro305Arg | Exon 9 | pathogenic | probably damaging (1.000) | Damaging (0.000) | Yamaguchi (2006), Martín-Hernández (2014), Lu (2020) |
| 457 |  | c.916A>T | p.Arg306* | Exon 9 | NR | - | - | Shchelochkov (2009) |
| 458 |  | c.917G>C | p.Arg306Thr | Exon 9 | NR | probably damaging (1.000) | Damaging (0.000) | Meng (2013) |
| 459 |  | c.919A>G | p.Lys307Glu | Exon 9 | Conflicting interpretations of pathogenicity | possibly damaging (0.833) | Damaging (0.023) | Lu (2020) |
| 460 |  | c.928_930del | p.Glu310del | Exon 9 | uncertain significance | - | - | Tuchman (1995a) |
| 461 | rs72558466 | c.928G>T | p.Glu310* | Exon 9 | pathogenic | - | - | Reish (1993), Giorgi (2000) |
| 462 |  | c.929_931del | p.Glu310Valfs*45 | Exon 9 | NR | - | - | Storkanova (2013), Lu (2020), Kido (2021) |
| 463 | rs72558467 | c.929A>G | p.Glu310Gly | Exon 9 | pathogenic | probably damaging (1.000) | Damaging (0.000) | Yamaguchi (2006), Shao (2017) |
| 464 | rs72558468 | c.931G>A | p.Val311Met | Exon 9 | pathogenic | probably damaging (1.000) | Damaging (0.000) | Yamaguchi (2006), Lu (2020) |
| 465 |  | c.932T>A | p.Val311Glu | Exon 9 | NR | probably damaging (1.000) | Damaging (0.000) | Martín-Hernández (2014) |
| 466 |  | c.940G>T | p.Glu314* | Exon 9 | NR | - | - | Kido (2021) |
| 467 | rs72558469 | c.941_943del | p.Glu314del | Exon 9 | pathogenic | - | - | Yamaguchi (2006) |
| 468 | rs72558470 | c.943G>T | p.Val315Phe | Exon 9 | likely pathogenic | probably damaging (0.997) | Damaging (0.000) | Yamaguchi (2006) |
| 469 | rs67414444 | c.944T>A | p.Val315Asp | Exon 9 | pathogenic | probably damaging (1.000) | Damaging (0.000) | Tuchman (2002) |
| 470 | rs67414444 | c.944T>G | p.Val315Gly | Exon 9 | pathogenic | probably damaging (1.000) | Damaging (0.000) | Tuchman (2002), Lu (2020) |
| 471 |  | c.944T>C | p.Val315Ala | Exon 9 | pathogenic | probably damaging (1.000) | Damaging (0.001) | Gobin-Limballe (2021) |
| 472 |  | c.946T>G | p.Phe316Val | Exon 9 | pathogenic | possibly damaging (0.921) | Damaging (0.000) | Gobin-Limballe (2021) |
| 473 | rs72558471 | c.947T>C | p.Phe316Ser | Exon 9 | pathogenic | probably damaging (1.000) | Damaging (0.000) | Tuchman (2002) |
| 474 | rs72558472 | c.953C>T | p.Ser318Phe | Exon 9 | pathogenic | benign (0.183) | Damaging (0.011) | Genet (2000) |
| 475 | rs72558473 | c.958C>T | p.Arg320* | Exon 9 | pathogenic | - | - | Yoo (1996), Matsuda (1997), Kim (2006), Choi (2015) |
| 476 | rs72558474 | c.959G>T | p.Arg320Leu | Exon 9 | likely pathogenic | probably damaging (0.999) | Damaging (0.002) | Tuchman (1995a) |
| 477 | rs72558475 | c.962C>A | p.Ser321* | Exon 9 | pathogenic | - | - | Tuchman (2002) |
| 478 |  | c.964C>G | p.Leu322Val | Exon 9 | NR | possibly damaging (0.643) | Tolerated (0.156) | Caldovic (2015) |
| 479 |  | c.965T>C | p.Leu322Pro | Exon 9 | NR | probably damaging (1.000) | Damaging (0.000) | Shchelochkov (2009) |
| 480 |  | c.967G>A | p.Val323Met | Exon 9 | NR | probably damaging (0.995) | Damaging (0.001) | Kim (2006), Choi (2015) |
| 481 |  | c.970_979delTTCCCAGAGG | p.Phe324Glnfs*16 | Exon 9 | likely pathogenic | - | - | Wang (2022) |
| 482 | rs72558476 | c.976G>A | p.Glu326Lys | Exon 9 | pathogenic | probably damaging (0.994) | Damaging (0.001) | Popowska (1999) |
| 483 | rs72558477 | c.982G>T | p.Glu328* | Exon 9 | pathogenic | - | - | Yamaguchi (2006) |
| 484 | rs72558478 | c.988A>G | p.Arg330Gly | Exon 9 | likely pathogenic | probably damaging (1.000) | Damaging (0.000) | Tuchman (1997) |
| 485 |  | c.988_990delAGAinsT | p.Arg330* | Exon 9 | NR | - | - | Climent (2002) |
| 486 | rs72558479 | c.991A>T | p.Lys331* | Exon 9 | pathogenic | - | - | Yamaguchi (2006) |
| 487 | rs72558480 | c.994T>A | p.Trp332Arg | Exon 9 | pathogenic | probably damaging (1.000) | Damaging (0.002) | Rapp (2001) |
| 488 | rs72558481 | c.995G>A | p.Trp332* | Exon 9 | pathogenic | - | - | Yamaguchi (2006) |
| 489 |  | c.995G>C | p.Trp332Ser | Exon 9 | NR | probably damaging (1.000) | Damaging (0.001) | Wang (2014) |
| 490 | rs72558482 | c.996G>A | p.Trp332* | Exon 9 | pathogenic | - | - | Matsuura (1994) |
| 491 |  | c.997A>G | p.Thr333Ala | Exon 9 | NR | probably damaging (0.982) | Tolerated (0.768) | Climent (2002) |
| 492 | rs281865553 | c.1005G>A | p.Met335Ile | Exon 9 | pathogenic | possibly damaging (0.900) | Tolerated (0.478) | Tuchman (2002), Ali (2018) |
| 493 |  | c.1005+1G>A | - | Intron 9 | NR | - | - | Shao (2017) |
| 494 | rs72558483 | c.1005+1G>T | - | Intron 9 | pathogenic | - | - | Tuchman (1997) |
| 495 | rs72558484 | c.1005+2T>C | - | Intron 9 | pathogenic | - | - | Tuchman (2002) |
| 496 |  | c.1005+1091C>G | - | Intron 9 | NR | - | - | Engel (2008) |
| 497 | rs67916658 | c.1006-1G>A | - | Intron 9 | pathogenic | - | - | Gyato (2004) |
| 498 | rs72558485 | c.1006-3C>G | - | Intron 9 | pathogenic | - | - | Climent (2002) |
| 499 | rs72558486 | c.1006G>T | p.Ala336Ser | Exon 10 | pathogenic | possibly damaging (0.544) | Tolerated (0.376) | Tuchman (1998) |
| 500 |  | c.1009G>T | p.Val337Phe | Exon 10 | pathogenic | probably damaging (0.966) | Damaging (0.003) | Gobin-Limballe (2021) |
| 501 | rs72558487 | c.1009G>C | p.Val337Leu | Exon 10 | pathogenic | benign (0.002) | Tolerated (0.150) | Matsuda (1997) |
| 502 |  | c.1015G>A | p.Val339Met | Exon 10 | Pathogenic | possibly damaging (0.843) | Damaging (0.021) | Kido (2021), Gobin-Limballe (2021) |
| 503 | rs72558488 | c.1015G>C | p.Val339Leu | Exon 10 | pathogenic | benign (0.000) | Tolerated (0.063) | Tuchman (1997) |
| 504 |  | c.1016T>G | p.Val339Gly | Exon 10 | NR | probably damaging (0.991) | Tolerated (0.065) | Wang (2014) |
| 505 | rs72558489 | c.1018T>C | p.Ser340Pro | Exon 10 | pathogenic | probably damaging (0.979) | Damaging (0.016) | Oppliger Leibundgut (1997), Storkanova (2013) |
| 506 |  | c.1019C>T | p.Ser340Phe | Exon 10 | Conflicting interpretations of pathogenicity | probably damaging (0.994) | Damaging (0.003) | Lu (2020) |
| 507 | rs72558490 | c.1022T>C | p.Leu341Pro | Exon 10 | pathogenic | probably damaging (1.000) | Damaging (0.004) | Climent (2002) |
| 508 |  | c.1025T>G | p.Leu342Pro | Exon 10 | NR | probably damaging (1.000) | Damaging (0.002) | Bernal (2021) |
| 509 | rs72558491 | c.1028C>A | p.Thr343Lys | Exon 10 | pathogenic | benign (0.020) | Tolerated (0.154) | Tuchman (1995a), Oppliger Leibundgut (1996a) |
| 510 |  | c.1028C>G | p.Thr343Arg | Exon 10 | uncertain significance | possibly damaging (0.754) | Damaging (0.037) | Martín-Hernández (2014), Caldovic (2015) |
| 511 | rs66469337 | c.1033T>C | p.Tyr345His | Exon 10 | uncertain significance | benign (0.321) | Tolerated (0.238) | Yamaguchi (2006) |
| 512 | rs66469337 | c.1033T>G | p.Tyr345Asp | Exon 10 | pathogenic | probably damaging (0.999) | Damaging (0.007) | Tuchman (1995a) |
| 513 | rs72558492 | c.1034A>G | p.Tyr345Cys | Exon 10 | pathogenic | probably damaging (0.999) | Damaging (0.003) | Tuchman (1998), Caldovic (2015) |
| 514 | rs72558493 | c.1039C>A | p.Pro347Thr | Exon 10 | pathogenic | probably damaging (0.984) | Damaging (0.021) | Yamaguchi (2006) |
| 515 |  | c.1039C>T | p.Pro347Ser | Exon 10 | NR | probably damaging (0.999) | Damaging (0.048) | Caldovic (2015) |
| 516 |  | c.1040C>T | p.Pro347Leu | Exon 10 | NR | probably damaging (0.996) | Damaging (0.009) | Caldovic (2015) |
| 517 | rs72558494 | c.1042C>T | p.Gln348* | Exon 10 | pathogenic | - | - | Oppliger Leibundgut (1997) |
| 518 |  | c.1043delA | p.Gln348Argfs*47 | Exon 10 | NR | - | - | Storkanova (2013) |
| 519 |  | c.1046T>C | p.Leu349Pro | Exon 10 | NR | probably damaging (0.999) | Damaging (0.004) | Caldovic (2015) |
| 520 |  | c.1052delA | p.Lys351Serfs*44 | Exon 10 | pathogenic | - | - | Gobin-Limballe (2021) |
| 521 | rs72558495 | c.1061T>G | p.Phe354Cys | Exon 10 | likely pathogenic | probably damaging (1.000) | Damaging (0.001) | Tuchman (1997) |
| 522 |  | c.1063T>C | p.*355Glu | Exon 10 | NR | - | - | Caldovic (2015) |
| 523 |  | c.1065A>T | p.*355Cysext*14 | Exon 10 | NR | - | - | Storkanova (2013) |
