# Supplementary material for: Pathogenic variants of ornithine transcarbamylase deficiency: Nation-wide study in Japan and literature review
Source: Front Genet. 2022 Oct 11;13:952467. doi: 10.3389/fgene.2022.952467 (PMC9593096; doi:10.3389/fgene.2022.952467)
Supplement: Supplementary file 5 [file DataSheet1.docx]

Supplemental data 1. Variants in the *OTC* gene and phenotype

The variants were categorized by protein level descriptions.

**Substitution** (Missense variant)

| Variant No. | Nucleic acid | Amino acid | Location | Phenotype (onset-time) | NH3 (μmol/L) | References |
| --- | --- | --- | --- | --- | --- | --- |
| 2 | c.1A>G | p.Met1Val | Ex 1 | F (12y) | NA | Oppliger Leibundgut (1997) |
| 3 | c.1A>T | p.Met1Leu | Ex 1 | N (NA) | NA | Yamaguchi (2006) |
| 4 | c.2T>C | p.Met1Thr | Ex 1 | F (NA) | NA | Yamaguchi (2006) |
| 5 | c.3G>A | p.Met1Ile | Ex 1 | F (21y) | NA | Climent (2002) |
|  |  |  |  | F (6y) | 194 | Lu (2020) |
| 9 | c.34G>A | p.Ala12Thr | Ex 1 | F (7y) | 284 | Tanaka (2005) |
| 13 | c.77G>A | p.Arg26Gln | Ex 1 | N (14d) | NA | Grompe (1989) |
|  |  |  |  | N (5d) | 1,260 | Kim (2006) |
|  |  |  |  | N (10d) | 735 | Choi (2015) |
|  |  |  |  | N (11d) | 1,365 | Shao (2017) |
|  |  |  |  | N (2d) | 400 | Zheng (2020) |
|  |  |  |  | N (5d) | 2,353 | Kido (2021) |
|  |  |  |  | F (2.3y) | 213 | Choi (2015) |
| 14 | c.77G>C | p.Arg26Pro | Ex 1 | N (NA) | NA | Yamaguchi (2006) |
|  |  |  |  | N (1m) | 280 | Kim (2006) |
|  |  |  |  | N (5d) | 511 | Choi (2015) |
| 30 | c.115G>T | p.Gly39Cys | Ex 2 | L (NA) | NA | Calvas (1998) |
| 31 | c.116G>A | p.Gly39Asp | Ex 2 | NA | NA | Shchelochkov (2009) |
| 32 | c.116G>T | p.Gly39Val | Ex 2 | N (1d) | >280 | Zheng (2020) |
|  |  |  |  | F (7m) | >280 | Zheng (2020) |
| 33 | c.116G>C | p.Gly39Ala | Ex 2 | L (NA) | NA | Gobin-Limballe (2021) |
| 34 | c.118C>T | p.Arg40Cys | Ex 2 | L (NA) | NA | Oppliger Leibundgut (1995) |
| 35 | c.119G>A | p.Arg40His | Ex 2 | N (7d) | 310 | Zhou (2020) |
|  |  |  |  | L (NA) | NA | Tuchman (1994a) |
|  |  |  |  | L (10y11m) | 212 | Nishiyori (1997) |
|  |  |  |  | L (17y) | 134 | Nishiyori (1997) |
|  |  |  |  | L (11y) | 33 | Takanashi (2002) |
|  |  |  |  | L (44y) | 845 | Cavicchi (2014) |
|  |  |  |  | L (12y) | 795 | Shao (2017) |
|  |  |  |  | L (56y) | 1,152 | Lu (2020) |
|  |  |  |  | F (52y) | 402 | Kido (2021) |
|  |  |  |  | F (30y) | 109 | This study |
|  |  |  |  | F (asymptomatic) | 17 | This study |
| 36 | c.119G>T | p.Arg40Leu | Ex 2 | L (45y) | 411 | Cavicchi (2014) |
| 37 | c.121G>T | p.Asp41Tyr | Ex 2 | F (1y2m) | 270 | Lee (2020) |
| 38 | c.122A>G | p.Asp41Gly | Ex 2 | F (NA) | NA | Yamaguchi (2006) |
| 39 | c.122A>C | p.Asp41Ala | Ex 2 | L (1.1y) | 300 | Lu (2020) |
| 42 | c.127C>T | p.Leu43Phe | Ex 2 | F (NA) | NA | Oppliger Leibundgut (1997) |
| 43 | c.128T>C | p.Leu43Pro | Ex 2 | N/F (NA) | NA | Gobin-Limballe (2021) |
| 44 | c.131C>T | p.Thr44Ile | Ex 2 | F (16m) | 424 | Yoo (1996) |
|  |  |  |  | F (2m) | 389 | Kim (2006) |
| 45 | c.133C>G | p.Leu45Val | Ex 2 | N (2d) | 850 | Ali (2018) |
|  |  |  |  | F (NA) | NA | Tuchman (1998) |
| 46 | c.134T>C | p.Leu45Pro | Ex 2 | N (NA) | NA | Grompe (1989) |
| 48 | c.140A>C | p.Asn47Thr | Ex 2 | F (NA) | NA | Yamaguchi (2006) |
| 49 | c.140A>T | p.Asn47Ile | Ex 2 | N (NA) | NA | Tuchman (1997) |
|  |  |  |  | F (8y) | 285 | Choi (2015) |
|  |  |  |  | F (9y) | 91 | Kido (2021) |
| 52 | c.142T>G | p.Phe48Val | Ex 2 | L (1y1m) | 405 | Kido (2021) |
| 53 | c.143T>C | p.Phe48Ser | Ex 2 | F (NA) | NA | Genet (2000) |
| 55 | c.145A>C | p.Thr49Pro | Ex 2 | F (NA) | NA | Yamaguchi (2006) |
| 56 | c.148G>A | p.Gly50Arg | Ex 2 | L (NA) | NA | Tuchman (1997) |
| 58 | c.154G>A | p.Glu52Lys | Ex 2 | N (NA) | NA | McCullough (2000) |
|  |  |  |  | F (NA) | NA | Martín-Hernández (2014) |
| 60 | c.155A>G | p.Glu52Gly | Ex 2 | N (NA) | NA | Yamaguchi (2006) |
| 61 | c.156A>T | p.Glu52Asp | Ex 2 | L (NA) | NA | McCullough (2000) |
| 62 | c.158T>C | p.Ile53Thr | Ex 2 | L (NA) | NA | Yamaguchi (2006) |
| 63 | c.158T>G | p.Ile53Ser | Ex 2 | N (NA) | NA | Tuchman (2002) |
| 64 | c.163T>G | p.Tyr55Asp | Ex 2 | L (46y) | NA | Nishiyori (1998) |
| 65 | c.167T>C | p.Met56Thr | Ex 2 | L (NA) | NA | Tuchman (1997) |
| 66 | c.170T>A | p.Leu57Gln | Ex 2 | N (NA) | NA | Yamaguchi (2006) |
| 67 | c.170T>C | p.Leu57Pro | Ex 2 | F (2.8y) | 284 | Choi (2015) |
| 69 | c.176T>C | p.Leu59Pro | Ex 2 | N (2d) | 1,367 | Zhou (2020) |
| 70 | c.176T>G | p.Leu59Arg | Ex 2 | L (NA) | NA | Azevedo (2006) |
| 71 | c.179C>T | p.Ser60Leu | Ex 2 | F (2y) | NA | Tuchman (1995a) |
|  |  |  |  | L (8m) | NA | Kido (2021) |
| 72 | c.184G>C | p.Asp62His | Ex 2 | NA (NA) | NA | Shchelochkov (2009) |
| 73 | c.185A>G | p.Asp62Gly | Ex 2 | F (NA) | NA | Caldovic (2015) |
| 74 | c.188T>C | p.Leu63Pro | Ex 2 | F (30m) | NA | Oppliger Leibundgut (1997) |
| 75 | c.196A>G | p.Arg66Gly | Ex 2 | N (5d) | 520 | Kido (2021) |
| 76 | c.200T>G | p.Ile67Arg | Ex 2 | F (NA) | NA | Yamaguchi (2006) |
| 86 | c.227T>C | p.Leu76Ser | Ex 3 | N (NA) | NA | Genet (2000) |
|  |  |  |  | F (NA) | 250 | Kido (2021) |
| 87 | c.231G>T | p.Leu77Phe | Ex 3 | L (NA) | NA | McCullough (2000) |
| 88 | c.231G>C | p.Leu77Phe | Ex 3 | L (15y) | 156 | Shao (2017) |
| 90 | c.236G>A | p.Gly79Glu | Ex 3 | N (5d) | 1,300 | Tuchman (1992) |
| 91 | c.238A>G | p.Lys80Glu | Ex 3 | F (3.5y) | 123 | Schultz (2000) |
| 92 | c.240G>T | p.Lys80Asn | Ex 3 | L (13y) | 750 | Galloway (2000) |
| 97 | c.247G>C | p.Gly83Arg | Ex 3 | N (NA) | NA | Tuchman (1997) |
| 98 | c.248G>A | p.Gly83Asp | Ex 3 | N (NA) | NA | Bartholomew (1998) |
| 99 | c.254T>A | p.Ieu85Gln | Ex 3 | F (7y1m) | 119 | Kido (2021) |
| 100 | c.254T>G | p.Ile85Ser | Ex 3 | L (NA) | NA | Martín-Hernández (2014) |
| 102 | c.259G>A | p.Glu87Lys | Ex 3 | L (7.5m) | >700 | Tuchman (1995a) |
| 103 | c.263A>C | p.Lys88Thr | Ex 3 | L (NA) | NA | Martín-Hernández (2014) |
| 104 | c.264A>T | p.Lys88Asn | Ex 3 | L (8m) | NA | Reish (1993) |
|  |  |  |  | L (9m) | 200 | Tuchman (1995) |
|  |  |  |  | L (7y) | 215 | Oppliger Leibundgut (1996a) |
| 105 | c.268A>G | p.Ser90Gly | Ex 3 | F (7y) | 150 | Takanashi (2002) |
| 106 | c.269G>A | p.Ser90Asn | Ex 3 | N (NA) | NA | McCullough (2000) |
| 107 | c.270T>G | p.Ser90Arg | Ex 3 | F (NA) | NA | Tuchman (1998) |
|  |  |  |  | F (4y) | 282 | Shao (2017) |
| 110 | c.274C>G | p.Arg92Gly | Ex 3 | F (NA) | NA | Yamaguchi (2006) |
|  |  |  |  | L (63y) | 1,234 | Ramanatha (2017) |
| 111 | c.275G>A | p.Arg92Gln | Ex 3 | N (NA) | NA | Tuchman (1995a) |
|  |  |  |  | F (4y8m) | 201 | Schultz (2000) |
|  |  |  |  | F (1.5y) | 103 | Lu (2020) |
|  |  |  |  | F (10m) | 221 | This study |
| 112 | c.275G>T | p.Arg92Leu | Ex 3 | N (NA) | NA | Yamaguchi (2006) |
| 113 | c.275G>C | p.Arg92Pro | Ex 3 | N (NA) | NA | Yamaguchi (2006) |
| 114 | c.277A>G | p.Thr93Ala | Ex 3 | L (5m) | 379 | Tuchman (1995a) |
| 115 | c.278C>T | p.Thr93Ile | Ex 3 | F (NA) | NA | Martín-Hernández (2014) |
| 116 | c.281G>C | p.Arg94Thr | Ex 3 | L (7y) | 780 | Tuchman (1995a) |
| 117 | c.284T>C | p.Leu95Ser | Ex 3 | L (NA) | NA | McCullough (2000) |
| 118 | c.286T>C | p.Ser96Pro | Ex 3 | N (2d) | 865 | Ali (2018) |
| 119 | c.287C>T | p.Ser96Phe | Ex 3 | F (18m) | NA | Arranz (2007) |
| 120 | c.292G>A | p.Glu98Lys | Ex 3 | F (26m) | NA | Bisanzi (2002) |
| 122 | c.298G>T | p.Gly100Cys | Ex 3 | N (NA) | NA | Gobin-Limballe (2021) |
| 123 | c.298G>C | p.Gly100Asp | Ex 3 | L (10m) | 223 | Kim (2006) |
| 132 | c.299G>A | p.Gly100Asp | Ex 4 | N (5d) | 2,361 | Ali (2018) |
|  |  |  |  | F (3y) | NA | Oppliger Leibundgut (1997) |
| 133 | c.304G>C | p.Ala102Pro | Ex 4 | N (NA) | 1,355 | Storkanova (2013) |
|  |  |  |  | L (NA) | 169 | Kido (2021) |
| 134 | c.305C>A | p.Ala102Glu | Ex 4 | N (<72h) | 277 | Tuchman (1995a) |
| 136 | c.314G>A | p.Gly105Glu | Ex 4 | L (21y) | 377 | Cavicchi (2014) |
| 137 | c.314G>T | p.Gly105Val | Ex 4 | F (NA) | NA | Yamaguchi (2006) |
| 138 | c.316G>A | p.Gly106Arg | Ex 4 | F (NA) | NA | McCullough (2000) |
| 139 | c.317G>A | p.Gly106Glu | Ex 4 | F (1y) | >400 | Takanashi (2002) |
| 140 | c.317G>T | p.Gly106Val | Ex 4  Ex 4 | F (NA) | NA | Yamaguchi (2006) |
|  |  |  |  | F (2.3y) | 1,300 | Lu (2020) |
| 141 | c.325T>C | p.Cys109Arg | Ex 4 | N (1d) | 700 | Kido (2021) |
| 143 | c.327T>C | p.Cys109Arg | Ex 4 | F (NA) | NA | Caldovic (2015) |
| 145 | c.332T>C | p.Leu111Pro | Ex 4 | NA | NA | Grompe (1989) |
| 147 | c.350A>T | p.His117Leu | Ex 4 | L (NA) | NA | Tuchman (1995a) |
| 148 | c.350A>G | p.His117Arg | Ex 4 | L (10m | NA | Matsuda (1997) |
| 149 | c.350A>C | p.His117Pro | Ex 4 | L (3y6m) | 108 | Kido (2021) |
| 152 | c.365A>G | p.Glu122Gly | Ex 4 | L (13m) | NA | Arranz (2007) |
| 153 | c.365A>T | p.Glu122Val | Ex 4 | F (12m) | 1,100 | Nguyen (2020) |
| 154 | c.374C>T | p.Thr125Met | Ex 4 | N (14d) | NA | Gilbert-Dussardier (1996) |
| 156 | c.377A>G | p.Asp126Gly | Ex 4 | N (2d) | NA | Matsuura (1994) |
| 157 | c.385C>T | p.Arg129Cys | Ex 4 | NA | NA | Shchelochkov (2009) |
| 158 | c.386G>A | p.Arg129His | Ex 4 | N (4d) | NA | Garcia-Perez (1995a) |
|  |  |  |  | N (NA) | NA | Genet (2000) |
|  |  |  |  | N (NA) | NA | Rivera-Barahona (2015) |
|  |  |  |  | N (7d) | 300 | Lu (2020) |
|  |  |  |  | L (9m) | NA | Matsuura (1994) |
|  |  |  |  | L (3.5y) | NA | Garcia-Perez (1995a) |
|  |  |  |  | L (NA) | NA | Rivera-Barahona (2015) |
|  |  |  |  | L (8m) | 800 | Storkanova (2013) |
|  |  |  |  | L (14m) | 200 | Lee (2014) |
|  |  |  |  | L (9m) | 170 | Shao (2017) |
|  |  |  |  | L (1y) | 336 | Shao (2017) |
|  |  |  |  | L 2y) | 160 | Shao (2017) |
|  |  |  |  | L (2y4m) | 137 | Lee (2020) |
|  |  |  |  | L (NA) | 265 | Kido (2021) |
|  |  |  |  | L (1y2m) | 155 | This study |
| 159 | c.386G>C | p.Arg129Pro | Ex 4 | F (NA) | NA | Yamaguchi (2006) |
| 160 | c.386G>T | p.Arg129Leu | Ex 4 | L (NA) | NA | Strautnieks (1993) |
|  |  |  |  | L (8m) | 300 | Lu (2020) |
| 172 | c.392T>C | p.Leu131Ser | Ex 5 | L (NA) | NA | Yamaguchi (2006) |
|  |  |  |  | L (2y3m) | 278 | Kido (2021) |
| 173 | c.394T>C | p.Ser132Pro | Ex 5 | L (3y) | NA | Bisanzi (2002) |
| 174 | c.395C>T | p.Ser132Phe | Ex 5 | L (NA) | NA | Gyato (2004) |
| 176 | c.404C>A | p.Ala135Glu | Ex 5 | L (NA) | NA | Yamaguchi (2006) |
| 177 | c.407A>T | p.Asp136Val | Ex 5 | L (NA) | NA | Yamaguchi (2006) |
| 178 | c.409G>A | p.Ala137Thr | Ex 5 | F (NA) | NA | Yamaguchi (2006) |
| 179 | c.409G>C | p.Ala137Pro | Ex 5 | F (NA) | NA | Azevedo (2006) |
| 180 | c.416T>C | p.Leu139Ser | Ex 5 | F (NA) | NA | Tuchman (1997) |
| 181 | c.418G>C | p.Ala140Pro | Ex 5 | N (NA) | NA | Yamaguchi (2006) |
|  |  |  |  | F (8m) | 340 | Kim (2006) |
|  |  |  |  | F (2.6y) | 200 | Choi (2015) |
| 182 | c.419C>A | p.Ala140Asp | Ex 5 | NA | NA | Shchelochkov (2009) |
| 184 | c.421C>G | p.Arg141Gly | Ex 5 | F (NA) | NA | Yamaguchi (2006) |
|  |  |  |  | F (NA) | 59 | Lu (2020) |
| 185 | c.422G>A | p.Arg141Gln | Ex 5 | N (<72h) | <2,000 | Tuchman (1995a) |
|  |  |  |  | N (3d) | NA | Yoo (1996) |
|  |  |  |  | N (3d) | 487 | Kim (2006) |
|  |  |  |  | N (7d) | 158 | Kim (2006) |
|  |  |  |  | N (3d) | 2,915 | Ali (2018) |
|  |  |  |  | N (3d) | 4,276 | This study |
|  |  |  |  | N/F (7d) | 480 | Ali (2018) |
|  |  |  |  | L (12y) | 257 | Maddalena (1988) |
|  |  |  |  | L (7y) | 547 | Kim (2006) |
|  |  |  |  | L (3.4y) | 308 | Choi (2015) |
|  |  |  |  | L (2.3y) | 120 | Lu (2020) |
|  |  |  |  | L (1y7m) | 1,735 | Kido (2021) |
| 186 | c.422G>C | p.Arg141Pro | Ex 5 | F (NA) | NA | Tuchman (1997) |
| 187 | c.425T>A | p.Val142Glu | Ex 5 | L (NA) | NA | Tuchman (2002) |
| 191 | c.443T>C | p.Leu148Ser | Ex 5 | N (NA) | NA | Yamaguchi (2006) |
| 192 | c.443T>G | p.Leu148Trp | Ex 5 | F (NA) | NA | McCullough (2000) |
| 193 | c.444G>C | p.Leu148Phe | Ex 5 | F (2y) | NA | Komaki (1997) |
| 194 | c.444G>T | p.Leu148Phe | Ex 5 | F (NA) | NA | Matsuura (1998) |
| 196 | c.452T>G | p.Leu151Arg | Ex 5 | F (NA) | NA | Yamaguchi (2006) |
| 197 | c.455C>T | p.Ala152Val | Ex 5 | L (NA) | NA | Kogo (1998) |
| 200 | c.463G>C | p.Ala155Pro | Ex 5 | F (NA) | NA | Yamaguchi (2006) |
| 201 | c.463G>T | p.Ala155Ser | Ex 5 | F (NA) | NA | Tuchman (2002) |
| 202 | c.464C>A | p.Ala155Glu | Ex 5 | N (NA) | NA | Yamaguchi (2006) |
| 203 | c.472C>T | p.Pro158Ser | Ex 5 | L (1y) | 260 | Storkanova (2013) |
| 204 | c.473C>T | p.Pro158Leu | Ex 5 | N (NA) | NA | Gobin-Limballe (2021) |
| 205 | c.476T>C | p.Ile159Thr | Ex 5 | F (7y) | NA | Garcia-Perez (1995b) |
|  |  |  |  | F (NA) | NA | Martín-Hernández (2014) |
| 206 | c.477T>G | p.Ile159Met | Ex 5 | L (49y) | 541 | Ben-Ari (2010) |
| 207 | c.479T>A | p.Ile160Asn | Ex 5 | N (NA) | NA | Yamaguchi (2006) |
| 208 | c.479T>C | p.Ile160Thr | Ex 5 | N (NA) | NA | Yamaguchi (2006) |
| 209 | c.479T>G | p.Ile160Ser | Ex 5 | F (2y) | NA | Climent (2002) |
| 210 | c.481A>G | p.Asn161Asp | Ex 5 | N (NA) | NA | Genet (2000) |
|  |  |  |  | L (NA) | NA | Martín-Hernández (2014) |
| 211 | c.482A>G | p.Asn161Ser | Ex 5 | N (NA) | NA | Tuchman (1995b) |
|  |  |  |  | N (3d) | 346 | Lu (2020) |
|  |  |  |  | F (2y) | <400 | Tuchman (1995a) |
|  |  |  |  | F (NA) | NA | Bisanzi (2002) |
|  |  |  |  | F (14y) | 429 | Kim (2006) |
|  |  |  |  | F (1y) | 564 | Shao (2017) |
|  |  |  |  | F (4y) | 153 | Chongsrisawat (2018) |
| 212 | c.483T>A | p.Asn161Lys | Ex 5 | F (9m) | 461 | Takanashi (2002) |
| 213 | c.484G>A | p.Gly162Arg | Ex 5 | N (2d) | 700 | Feldmann (1992) |
|  |  |  |  | N (<72h) | >700 | Tuchman (1995a) |
| 214 | c.484G>C | p.Gly162Arg | Ex 5 | F (NA) | NA | Yamaguchi (2006) |
| 215 | c.485G>A | p.Gly162Glu | Ex 5 | N (NA) | NA | Yamaguchi (2006) |
| 216 | c.488T>G | p.Leu163Arg | Ex 5 | F (NA) | NA | Gobin-Limballe (2021) |
| 217 | c.490T>C | p.Ser164Pro | Ex 5 | N (NA) | NA | Yamaguchi (2006) |
| 219 | c.493G>T | p.Asp165Tyr | Ex 5 | L (NA) | NA | Genet (2000) |
| 222 | c.503A>C | p.His168Pro | Ex 5 | L (NA) | NA | Yamaguchi (2006) |
| 223 | c.503A>G | p.His168Arg | Ex 5 | F (14m) | 235 | Vella (1996) |
| 224 | c.504T>A | p.His168Gln | Ex 5 | L (NA) | NA | Tuchman (1997) |
| 225 | c.505C>T | p.Pro169Ser | Ex 5 | N (NA) | NA | Genet (2000) |
|  |  |  |  | N (3d) | 967 | Kido (2021) |
| 226 | c.505C>G | p.Pro169Ala | Ex 5 | F (NA) | NA | Tuchman (2002) |
| 227 | c.506C>A | p.Pro169His | Ex 5 | F (NA) | NA | Caldovic (2015) |
| 228 | c.506C>T | p.Pro169Leu | Ex 5 | N (NA) | NA | Genet (2000) |
| 229 | c.511C>G | p.Gln171Glu | Ex 5 | NA | NA | Shchelochkov (2009) |
| 230 | c.513G>T | p.Gln171His | Ex 5 | N (3d) | 780 | Ali (2018) |
| 231 | c.514A>T | p.Ile172Phe | Ex 5 | F (NA) | NA | Climent (1999) |
| 232 | c.515T>A | p.Ile172Asn | Ex 5 | L (10m) | 114 | Ogino (2007) |
|  |  |  |  | L (11m) | 286 | Kido (2021) |
| 234 | c.516C>G | p.Ile172Met | Ex 5 | N (8d) | NA | Matsuura (1994) |
|  |  |  |  | N (20d) | 500 | Shao (2017) |
| 235 | c.520G>C | p.Ala174Pro | Ex 5 | F (3y) | NA | Tsai (1993) |
|  |  |  |  | F (NA) | 300 | Tuchman (1995a) |
| 237 | c.524A>G | p.Asp175Gly | Ex 5 | L (NA) | NA | Genet (2000) |
| 238 | c.524A>T | p.Asp175Val | Ex 5 | F (NA) | NA | Tuchman (1997) |
| 239 | c.526T>C | p.Tyr176His | Ex 5 | N (NA) | NA | Tuchman (2002) |
| 240 | c.527A>G | p.Tyr176Cys | Ex 5 | L (2.5y) | NA | Oppliger Leibundgut (1996a) |
| 241 | c.527A>C | p.Tyr176Ser | Ex 5 | F (NA) | NA | Azevedo (2006) |
| 243 | c.530T>G | p.Leu177Arg | Ex 5 | N/F (NA) | 215.3 | Kido (2021) |
| 245 | c.533C>T | p.Thr178Met | Ex 5 | N (NA) | NA | Oppliger Leibundgut (1995) |
|  |  |  |  | F (NA) | NA | Bisanzi (2002) |
|  |  |  |  | F (2.5y) | 326 | Choi (2015) |
|  |  |  |  | F (8y2m) | 1,764.7 | Kido (2021) |
| 246 | c.535C>T | p.Leu179Phe | Ex 5 | N (7d) | NA | Arranz (2007) |
|  |  |  |  | L (17m) | 171 | Fantur (2013) |
|  |  |  |  | L (NA) | 208 | Kido (2021) |
| 247 | c.536T>C | p.Leu179Pro | Ex 5 | N (NA) | NA | Yamaguchi (2006) |
| 249 | c.539_540AG>CC | p.Gln180Pro | Ex 5 | N (NA) | NA | Hübler (2001) |
| 250 | c.540G>C | p.Gln180His | Ex 5 | N (5d) | >380 | Tuchman (1995a) |
|  |  |  |  | N (7d) | 290 | Zhou (2020) |
|  |  |  |  | L (2y) | NA | Shimadzu (1998) |
|  |  |  |  | F (2y) | 256 | Lu (2020) |
| 259 | c.542A>G | p.Glu181Gly | Ex 6 | N (NA) | NA | Tuchman (1998) |
| 260 | c.545A>T | p.His182Leu | Ex 6 | N (<72h) | NA | Tuchman (1995a) |
| 261 | c.547T>G | p.Tyr183Asp | Ex 6 | F (NA) | NA | Oppliger Leibundgut (1997) |
| 262 | c.548A>G | p.Tyr183Cys | Ex 6 | N (<72h) | NA | Tuchman (1995a) |
|  |  |  |  | N (NA) | 2,800 | Storkanova (2013) |
|  |  |  |  | N (3d) | 1,779 | Choi (2015) |
|  |  |  |  | F (23m) | 495 | Lee (2014) |
| 264 | c.557T>C | p.Leu186Pro | Ex 6 | F (NA) | NA | Azevedo (2006) |
| 267 | c.562G>C | p.Gly188Arg | Ex 6 | N (10d) | NA | Gilbert-Dussardier (1996) |
|  |  |  |  | L (2y) | 218 | Lu (2020) |
| 268 | c.563G>T | p.Gly188Val | Ex 6 | F (NA) | NA | Climent (1999) |
| 269 | c.563G>C | p.Gly188Ala | Ex 6 | NA | NA | Shchelochkov (2009) |
| 273 | c.571C>T | p.Leu191Phe | Ex 6 | L (4y) | NA | Climent (2002) |
| 274 | c.572T>G | p.Leu191Arg | Ex 6 | N (NA) | NA | Yamaguchi (2006) |
| 275 | c.576C>G | p.Ser192Arg | Ex 6 | N (2d) | 1,212 | Matsuura (1993) |
| 276 | c.577T>C | p.Trp193Arg | Ex 6 | F (NA) | NA | Yamaguchi (2006) |
| 277 | c.577T>G | p.Trp193Gly | Ex 6 | F (NA) | NA | Yamaguchi (2006) |
| 280 | c.579G>C | p.Trp193Cys | Ex 6 | NA | NA | Shchelochkov (2009) |
| 281 | c.581T>C | p.Ile194Thr | Ex 6 | L (NA) | NA | Caldovic (2015) |
| 282 | c.583G>A | p.Gly195Arg | Ex 6 | N (NA) | NA | Tuchman (1994a) |
|  |  |  |  | N (3d) | 3,307 | Choi (2015) |
|  |  |  |  | N (2d) | 980 | Ali (2018) |
|  |  |  |  | N (3d) | 637 | Lu (2020) |
|  |  |  |  | N (1d) | 1,020 | Zhou (2020) |
|  |  |  |  | F (NA) | NA | Tuchman (1995a) |
|  |  |  |  | F (6m) | 387 | Lu (2020) |
|  |  |  |  | F (29y) | 421 | Lu (2020) |
|  |  |  |  | F (NA) | 353 | Kido (2021) |
| 283 | c.583G>C | p.Gly195Arg | Ex 6 | F (6y) | NA | Storkanova (2013) |
|  |  |  |  | F (12m) | 115 | Kim (2006) |
|  |  |  |  | F (0.7y) | 364 | Choi (2015) |
| 284 | c.584G>C | p.Gly195Ala | Ex 6 | N (NA) | NA | Gobin-Limballe (2021) |
| 286 | c.586G>A | p.Asp196Asn | Ex 6 | L (NA) | NA | Yamaguchi (2006) |
|  |  |  |  | L (10m) | 177 | Shao (2017) |
|  |  |  |  | L (6y) | 96 | Shao (2017) |
| 287 | c.586G>T | p.Asp196Tyr | Ex 6 | N (NA) | NA | Tuchman (1998) |
|  |  |  |  | F (NA) | NA | Azevedo (2006) |
| 288 | c.586G>C | p.Asp196His | Ex 6 | NA | NA | Lin (2010) |
| 289 | c.587A>T | p.Asp196Val | Ex 6 | N (5d) | 497 | Matsuura (1993) |
| 290 | c.589G>A | p.Gly197Arg | Ex 6 | F (NA) | NA | Climent (1999) |
| 291 | c.589G>T | p.Gly197Trp | Ex 6 | NA | NA | Shchelochkov (2009) |
| 292 | c.590G>A | p.Gly197Glu | Ex 6 | F (NA) | NA | Tuchman (1998) |
| 293 | c.593A>T | p.Asn198Ile | Ex 6 | N (NA) | NA | Yamaguchi (2006) |
| 294 | c.594C>A | p.Asn198Lys | Ex 6 | F (NA) | NA | Popowska (1999) |
| 295 | c.595A>G | p.Asn199Asp | Ex 6 | N (5d) | 990 | Ali (2018) |
|  |  |  |  | N (1d) | 180 | Liu (2021) |
|  |  |  |  | F (NA) | NA | Yamaguchi (2006) |
| 296 | c.595A>C | p.Asn199His | Ex 6 | F (6y) | 310 | Ali (2018) |
| 297 | c.596A>G | p.Asn199Ser | Ex 6 | N (NA) | NA | Tuchman (2002) |
| 299 | c.601C>A | p.Leu201Met | Ex 6 | F (1y) | 473 | Shao (2017) |
| 300 | c.602T>C | p.Leu201Pro | Ex 6 | N (1d) | NA | Shimadzu (1998) |
| 301 | c.604C>T | p.His202Tyr | Ex 6 | L (NA) | NA | Tuchman (1997) |
|  |  |  |  | L (4m) | 350 | Lu (2020) |
|  |  |  |  | F (14y) | 345 | Kido (2021) |
| 302 | c.605A>C | p.His202Pro | Ex 6 | F (NA) | NA | Staudt (1998) |
| 303 | c.605A>T | p.His202Leu | Ex 6 | L (NA) | NA | Martín-Hernández (2014) |
| 304 | c.607T>C | p.Ser203Pro | Ex 6 | N (NA) | 1,514 | Bernal (2021) |
|  |  |  |  | F (NA) | 300 | Bernal (2021) |
| 305 | c.608C>T | p.Ser203Phe | Ex 6 | N (NA) | NA | Gobin-Limballe (2021) |
| 306 | c.608C>G | p.Ser203Cys | Ex 6 | F (NA) | NA | Tuchman (1995a) |
| 307 | c.613A>G | p.Met205Val | Ex 6 | N (NA) | NA | Genet (2000) |
| 308 | c.614T>C | p.Met205Thr | Ex 6 | N (4d) | 980 | Kim (2006) |
|  |  |  |  | F (1y1m) | 301 | Kido (2021) |
| 309 | c.617T>G | p.Met206Arg | Ex 6 | N (NA) | NA | Tuchman (1997) |
|  |  |  |  | F (4y) | 195 | Kim (2006) |
|  |  |  |  | F (10m) | NA | Kido (2021) |
| 310 | c.618G>C | p.Met206Ile | Ex 6 | F (20m) | NA | Climent (2002) |
| 311 | c.620G>A | p.Ser207Asn | Ex 6 | N (NA) | NA | Yamaguchi (2006) |
|  |  |  |  | N (NA) | 1,100 | Storkanova (2013) |
| 312 | c.621C>A | p.Ser207Arg | Ex 6 | N (3d) | NA | Shimadzu (1998) |
| 313 | c.622G>A | p.Ala208Thr | Ex 6 | L (NA) | NA | Diggelen (2008) |
|  |  |  |  | L (5y) | 906 | Schultz (2000) |
|  |  |  |  | L (66y) | 1,145 | Cavicchi (2014) |
|  |  |  |  | L (69y) | 231 | Daijo (2017) |
|  |  |  |  | L (36y) | 427 | Lu (2020) |
|  |  |  |  | F (NA) | NA | Bisanzi (2002) |
| 314 | c.626C>A | p.Ala209Glu | Ex 6 | F (32y) | 173 | Bailly (2015) |
| 315 | c.626C>T | p.Ala209Val | Ex 6 | N (3d) | NA | Gilbert-Dussardier (1996) |
|  |  |  |  | N (2d) | 704 | Zhou (2020) |
|  |  |  |  | N (3d) | 229 | Zhou (2020) |
|  |  |  |  | F (11m) | NA | Garcia-Perez (1995b) |
|  |  |  |  | F (9m) | >400 | Takanashi (2002) |
|  |  |  |  | F (6y) | 223 | Takanashi (2002) |
|  |  |  |  | F (1.5y) | 133 | Lu (2020) |
|  |  |  |  | F (2.3y) | 300 | Lu (2020) |
|  |  |  |  | F (3y) | 118 | Kido (2021) |
| 316 | c.628A>C | p.Lys210Gln | Ex 6 | N/F (NA) | 830 | Storkanova (2013) |
|  |  |  |  | N/F (60h) | 981 | Valik (2004) |
| 317 | c.628A>G | p.Lys210Glu | Ex 6 | F (8m) | 364 | Storkanova (2013) |
| 319 | c.630A>C | p.Lys210Asn | Ex 6 | F (NA) | NA | Azevedo (2006) |
| 320 | c.635G>T | p.Gly212Val | Ex 6 | N (NA) | NA | Gobin-Limballe (2021) |
| 321 | c.638T>A | p.Met213Lys | Ex 6 | F (12m) | NA | Oppliger Leibundgut (1997) |
| 322 | c.638T>G | p.Met213Arg | Ex 6 | NA | NA | Caldovic (2015) |
| 323 | c.638T>C | p.Met213Thr | Ex 6 | N (2d) | 976 | Ali (2018) |
|  |  |  |  | F (NA) | NA | Caldovic (2015) |
|  |  |  |  | F (14y) | NA | Ali (2018) |
|  |  |  |  | F (34y) | 250 | Ali (2018) |
| 324 | c.640C>T | p.His214Tyr | Ex 6 | N (3d) | 790 | Yoo (1996) |
|  |  |  |  | N (4d) | 790 | Kim (2006) |
|  |  |  |  | N (3d) | 3,157 | Choi (2015) |
| 326 | c.643C>T | p.Leu215Phe | Ex 6 | F (4y) | NA | Ueta (2001) |
|  |  |  |  | F (8m) | 180 | Kido (2021) |
| 327 | c.646C>G | p.Gln216Glu | Ex 6 | N (NA) | NA | Grompe (1989) |
| 328 | c.650C>A | p.Ala217Glu | Ex 6 | F (NA) | NA | Yamaguchi (2006) |
| 329 | c.652G>A | p.Ala218Thr | Ex 6 | F (7y9m) | 195 | This study |
| 330 | c.652G>C | p.Ala218Pro | Ex 6 | N (NA) | NA | Gobin-Limballe (2021) |
| 331 | c.653C>T | p.Ala218Val | Ex 6 | NA | NA | Shchelochkov (2009) |
| 332 | c.658C>A | p.Pro220Thr | Ex 6 | F (2y) | NA | Arranz (2007) |
| 333 | c.658C>G | p.Pro220Ala | Ex 6 | L (2.5y) | 1,176 | Oppliger Leibundgut (1996a) |
| 334 | c.659C>T | p.Pro220Leu | Ex 6 | N (NA) | NA | Yamaguchi (2006) |
| 336 | c.663G>T | p.Lys221Asn | Ex 6 | F (6y) | 215 | Kim (2006) |
| 337 | c.663G>C | p.Lys221Asn | Ex 6 | N (NA) | NA | Yamaguchi (2006) |
| 347 | c.673C>A | p.Pro225Thr | Ex 7 | L (8y) | 379 | Tuchman (1995a) |
| 348 | c.674C>G | p.Pro225Arg | Ex 7 | N (NA) | NA | Garcia-Perez (1995b) |
| 349 | c.674C>T | p.Pro225Leu | Ex 7 | N (72h) | 2,000 | Tuchman (1995a) |
|  |  |  |  | N (1d) | 1,877 | Shao (2017) |
|  |  |  |  | N (4d) | 1,048 | Shao (2017) |
| 350 | c.674_675CG>AA | p.Pro225Gln | Ex 7 | F (1y5m) | 268 | Kido (2021) |
| 352 | c.698C>T | p.Ala233Val | Ex 7 | N (NA) | NA | Yamaguchi (2006) |
| 355 | c.704A>C | p.Gln235Pro | Ex 7 | F (3y) | 286 | Lu (2020) |
| 356 | c.710C>A | p.Ala237Asp | Ex 7 | F (2y) | 135 | Kim (2006) |
| 357 | c.716A>T | p.Glu239Val | Ex 7 | N (NA) | NA | Yamaguchi (2006) |
| 358 | c.716A>G | p.Glu239Gly | Ex 7 | L (NA) | NA | Yamaguchi (2006) |
| 360 | c.717G>C | p.Glu239Asp | Ex 7 | F (NA) | NA | Yamaguchi (2006) |
| 369 | c.725C>T | p.Thr242Ile | Ex 8 | L (NA) | NA | Tuchman (1997) |
|  |  |  |  | N (9d) | 815 | Lu (2020) |
| 371 | c.731T>A | p.Leu244Gln | Ex 8 | L (NA) | NA | Calvas (1998) |
| 372 | c.740C>A | p.Thr247Lys | Ex 8 | N (<72h) | 2,000 | Tuchman (1995a) |
| 373 | c.740C>G | p.Thr247Arg | Ex 8 | F (NA) | NA | Gobin-Limballe (2021) |
| 374 | c.746A>G | p.Asp249Gly | Ex 8 | N (2d) | 116 | Kim (2006) |
|  |  |  |  | N (2d) | 166 | Choi (2015) |
| 375 | c.749C>T | p.Pro250Leu | Ex 8 | L (NA) | NA | Caldovic (2015) |
| 376 | c.757G>A | p.Ala253Thr | Ex 8 | N (NA) | NA | Yamaguchi (2006) |
| 377 | c.757G>C | p.Ala253Pro | Ex 8 | N (NA) | NA | Yamaguchi (2006) |
| 380 | c.764A>C | p.His255Pro | Ex 8 | F (NA) | NA | Tuchman (1998) |
| 383 | c.779T>C | p.Leu260Ser | Ex 8 | F (NA) | NA | Yamaguchi (2006) |
|  |  |  |  | F (1.1y) | 260 | Lu (2020) |
| 384 | c.782T>C | p.Ile261Thr | Ex 8 | N (NA) | 700 | Li (2018) |
| 386 | c.785C>A | p.Thr262Lys | Ex 8 | L (15m) | 109 | Giorgi (2000) |
| 387 | c.785C>T | p.Thr262Ile | Ex 8 | L (NA) | NA | Yamaguchi (2006) |
|  |  |  |  | L (9m) | 349 | Shao (2017) |
| 388 | c.787G>A | p.Asp263Asn | Ex 8 | F (NA) | NA | Tuchman (1997) |
| 389 | c.788A>G | p.Asp263Gly | Ex 8 | F (NA) | NA | Tuchman (1998) |
| 390 | c.790A>G | p.Thr264Ala | Ex 8 | L (4y) | 175 | Matsuura (1993) |
|  |  |  |  | L (4y) | NA | Kido (2021) |
|  |  |  |  | F (7m) | 192 | Giorgi (2000) |
| 391 | c.791C>A | p.Thr264Asn | Ex 8 | NA | NA | Hwu (2003b) |
| 392 | c.791C>T | p.Thr264Ile | Ex 8 | L (5y) | NA | Shimadzu (1998) |
| 393 | c.793T>C | p.Trp265Arg | Ex 8 | L (NA) | NA | Yamaguchi (2006) |
| 395 | c.794G>T | p.Trp265Leu | Ex 8 | L (12m) | 265 | Giorgi (2000) |
|  |  |  |  | L (4y) | 112 | Giorgi (2000) |
| 398 | c.799A>C | p.Ser267Arg | Ex 8 | F (8y) | NA | Shimadzu (1998) |
| 400 | c.802A>G | p.Met268Val | Ex 8 | NA | NA | Jamroz (2013) |
| 401 | c.803T>C | p.Met268Thr | Ex 8 | N (6d) | 1,030 | Zhou (2020) |
|  |  |  |  | L (6m) | 164 | Matsuura (1993) |
|  |  |  |  | F (NA) | NA | Bisanzi (2002) |
| 402 | c.805G>A | p.Gly269Arg | Ex 8 | L (11m) | 186 | Shao (2017) |
| 403 | c.806G>A | p.Gly269Glu | Ex 8 | N (10d) | 292 | Zimmer (1995) |
| 405 | c.809A>C | p.Gln270Pro | Ex 8 | F (NA) | NA | Yamaguchi (2006) |
| 410 | c.829C>T | p.Arg277Trp | Ex 8 | N (3d) | 700 | Lu (2020) |
|  |  |  |  | L (13m) | 406 | Hata (1991) |
|  |  |  |  | L (8m) | 150 | Matsuura (1993) |
|  |  |  |  | L (2y) | 400 | Kim (2006) |
|  |  |  |  | L (13y) | 400 | Storkanova (2013) |
|  |  |  |  | L (9y) | 197 | Storkanova (2013) |
|  |  |  |  | L (2y) | 148 | Storkanova (2013) |
|  |  |  |  | L (1.3y) | 71 | Choi (2015) |
|  |  |  |  | L (6y) | 202 | Shao (2017) |
|  |  |  |  | L (1.4y) | 351 | Lu (2020) |
|  |  |  |  | L (2.6y) | 244 | Lu (2020) |
|  |  |  |  | L (1.4y) | 114 | Lu (2020) |
|  |  |  |  | L (9.7y) | 120 | Lu (2020) |
|  |  |  |  | L (10m) | 131 | Lu (2020) |
|  |  |  |  | L (3y) | 289 | Lu (2020) |
|  |  |  |  | L (1y2m) | 557 | Lee (2020) |
|  |  |  |  | L (1y1m) | 559 | Kido (2021) |
|  |  |  |  | F (34y) | 901 | Cavicchi (2014) |
| 411 | c.830G>A | p.Arg277Gln | Ex 8 | L (8m) | 150 | Tuchman (1995a) |
| 412 | c.830G>T | p.Arg277Leu | Ex 8 | L (NA) | NA | Tuchman (2002) |
| 415 | c.842T>C | p.Phe281Ser | Ex 8 | N (7d) | 463 | Kim (2006) |
| 416 | c.850A>T | p.Tyr284Asn | Ex 8 | F (5y) | 1,926 | Chongsrisawat (2018) |
| 420 | c.859A>C | p.Thr287Pro | Ex 8 | F (NA) | NA | Caldovic (2015) |
| 421 | c.860C>T | p.Thr287Ile | Ex 8 | N (5d) | 50 | Lu (2020) |
| 424 | c.867G>T | p.Lys289Asn | Ex 8 | N (NA) | NA | Tuchman (2002) |
| 425 | c.867G>C | p.Lys289Asp | Ex 8 | N (NA) | NA | Caldovic (2015) |
| 435 | c.889G>T | p.Asp297Tyr | Ex 9 | NA | NA | Shchelochkov (2009) |
| 438 | c.892T>C | p.Trp298Arg | Ex 9 | F (NA) | NA | Caldovic (2015) |
| 439 | c.893G>C | p.Trp298Ser | Ex 9 | N (3d) | 565 | Ensenauer (2005) |
| 441 | c.902T>C | p.Leu301Ser | Ex 9 | F (NA) | NA | Caldovic (2015) |
| 442 | c.903A>T | p.Leu301Phe | Ex 9 | L (12y) | NA | Climent (2002) |
| 443 | c.904C>T | p.His302Tyr | Ex 9 | N (2d) | 780 | Oppliger Leibundgut (1996a) |
|  |  |  |  | N (1d) | >500 | Lu (2020) |
|  |  |  |  | F (3y6m) | 163 | Kido (2021) |
| 444 | c.905A>G | p.His302Arg | Ex 9 | N (NA) | NA | Genet (2000) |
| 445 | c.905A>T | p.His302Leu | Ex 9 | F (3y) | NA | Gilbert-Dussardier (1996) |
| 447 | c.906C>G | p.His302Gln | Ex 9 | L (NA) | NA | Tuchman (1997) |
| 448 | c.907T>C | p.Cys303Arg | Ex 9 | N (NA) | NA | Calvas (1998) |
| 449 | c.907T>G | p.Cys303Gly | Ex 9 | N (NA) | NA | Tuchman (2002) |
| 450 | c.908G>A | p.Cys303Tyr | Ex 9 | F (NA) | NA | Tuchman (1997) |
| 451 | c.912G>T | p.Leu304Phe | Ex 9 | L (7m) | 400 | Hoshide (1993) |
| 452 | c.912G>C | p.Leu304Phe | Ex 9 | L (4y) | NA | Matsuura (1995) |
|  |  |  |  | L (1y3m) | 169 | Kido (2021) |
| 453 | c.913C>T | p.Pro305Ser | Ex 9 | L (7m) | 392 | Lu (2020) |
| 454 | c.914C>A | p.Pro305His | Ex 9 | F (5y) | NA | Climent (2002) |
| 455 | c.914C>T | p.Pro305Leu | Ex 9 | F (1y) | 250 | Bernal (2021) |
| 456 | c.914C>G | p.Pro305Arg | Ex 9 | N (NA) | NA | Yamaguchi (2006) |
|  |  |  |  | F (NA) | NA | Martín-Hernández (2014) |
|  |  |  |  | F (1.8y) | 216 | Lu (2020) |
| 458 | c.917G>C | p.Arg306Thr | Ex 9 | NA | NA | Meng (2013) |
| 459 | c.919A>G | p.Lys307Glu | Ex 9 | L (1y) | 45 | Lu (2020) |
| 463 | c.929A>G | p.Glu310Gly | Ex 9 | L (NA) | NA | Yamaguchi (2006) |
|  |  |  |  | L (11m) | 100 | Shao (2017) |
| 464 | c.931G>A | p.Val311Met | Ex 9 | L (NA) | NA | Yamaguchi (2006) |
|  |  |  |  | L (1.2y) | 187 | Lu (2020) |
| 465 | c.932T>A | p.Val311Glu | Ex 9 | N (NA) | NA | Martín-Hernández (2014) |
| 468 | c.943G>T | p.Val315Phe | Ex 9 | F (NA) | NA | Yamaguchi (2006) |
| 469 | c.944T>A | p.Val315Asp | Ex 9 | F (NA) | NA | Tuchman (2002) |
| 470 | c.944T>G | p.Val315Gly | Ex 9 | F (NA) | NA | Tuchman (2002) |
|  |  |  |  | F (10m) | 315 | Lu (2020) |
| 471 | c.944T>C | p.Val315Ala | Ex 9 | N (NA) | NA | Gobin-Limballe (2021) |
| 472 | c.946T>G | p.Phe316Val | Ex 9 | L (NA) | NA | Gobin-Limballe (2021) |
| 473 | c.947T>C | p.Phe316Ser | Ex 9 | F (NA) | NA | Tuchman (2002) |
| 474 | c.953C>T | p.Ser318Phe | Ex 9 | F (NA) | NA | Genet (2000) |
| 476 | c.959G>T | p.Arg320Leu | Ex 9 | N (NA) | NA | Tuchman (1995a) |
| 478 | c.964C>G | p.Leu322Val | Ex 9 | F (NA) | NA | Caldovic (2015) |
| 479 | c.965T>C | p.Leu322Pro | Ex 9 | NA | NA | Shchelochkov (2009) |
| 480 | c.967G>A | p.Val323Met | Ex 9 | L (11m) | 182 | Kim (2006) |
|  |  |  |  | L (2.3y) | 227 | Choi (2015) |
| 482 | c.976G>A | p.Glu326Lys | Ex 9 | F (NA) | NA | Popowska (1999) |
| 484 | c.988A>G | p.Arg330Gly | Ex 9 | F (NA) | NA | Tuchman (1997) |
| 487 | c.994T>A | p.Trp332Arg | Ex 9 | N (NA) | NA | Rapp (2001) |
| 489 | c.995G>C | p.Trp332Ser | Ex 9 | N (NA) | NA | Wang (2014) |
| 491 | c.997A>G | p.Thr333Ala | Ex 9 | F (21y) | NA | Climent (2002) |
| 492 | c.1005G>A | p.Met335Ile | Ex 9 | N (NA) | NA | Tuchman (2002) |
|  |  |  |  | N (2d) | 1,065 | Ali (2018) |
| 499 | c.1006G>T | p.Ala336Ser | Ex 10 | L (NA) | NA | Tuchman (1998) |
| 500 | c.1009G>T | p.Val337Phe | Ex 10 | N (NA) | NA | Gobin-Limballe (2021) |
| 501 | c.1009G>C | p.Val337Leu | Ex 10 | L (6y) | NA | Matsuda (1997) |
| 502 | c.1015G>A | p.Val339Met | Ex 10 | N (3d) | 641.8 | Kido (2021) |
|  |  |  |  | F (NA) | NA | Gobin-Limballe (2021) |
| 503 | c.1015G>C | p.Val339Leu | Ex 10 | N (NA) | NA | Tuchman (1997) |
| 504 | c.1016T>G | p.Val339Gly | Ex 10 | N (NA) | NA | Wang (2014) |
| 505 | c.1018T>C | p.Ser340Pro | Ex 10 | N (NA) | 1,096 | Storkanova (2013) |
|  |  |  |  | F (4m) | NA | Oppliger Leibundgut (1997) |
| 506 | c.1019C>T | p.Ser340Phe | Ex 10 | L (8m) | 335 | Lu (2020) |
| 507 | c.1022T>C | p.Leu341Pro | Ex 10 | F (6m) | NA | Climent (2002) |
| 508 | c.1025T>G | p.Leu342Pro | Ex 10 | N/F (2d) | 146 | Bernal (2021) |
| 509 | c.1028C>A | p.Thr343Lys | Ex 10 | L (2y) | 200 | Oppliger Leibundgut (1996a) |
|  |  |  |  | F (8y) | 250 | Tuchman (1995a) |
| 510 | c.1028C>G | p.Thr343Arg | Ex 10 | L (NA) | NA | Martín-Hernández (2014) |
|  |  |  |  | N (NA) | NA | Caldovic (2015) |
| 511 | c.1033T>C | p.Tyr345His | Ex 10 | L (NA) | NA | Yamaguchi (2006) |
| 512 | c.1033T>G | p.Tyr345Asp | Ex 10 | F (3m) | 525 | Tuchman (1995a) |
| 513 | c.1034A>G | p.Tyr345Cys | Ex 10 | N (NA) | NA | Tuchman (1998) |
|  |  |  |  | F (NA) | NA | Caldovic (2015) |
| 514 | c.1039C>A | p.Pro347Thr | Ex 10 | F (NA) | NA | Yamaguchi (2006) |
| 515 | c.1039C>T | p.Pro347Ser | Ex 10 | N (NA) | NA | Caldovic (2015) |
| 516 | c.1040C>T | p.Pro347Leu | Ex 10 | F (NA) | NA | Caldovic (2015) |
| 519 | c.1046T>C | p.Leu349Pro | Ex 10 | F (NA) | NA | Caldovic (2015) |
|  |  |  |  | F (7y)mo | NA | Qin (2016) |
| 521 | c.1061T>G | p.Phe354Cys | Ex 10 | L (NA) | NA | Tuchman (1997) |

**No category** (Regulatory)

| Variant No. | Nucleic acid | Amino acid | Location | Phenotype (onset-time) | NH3 (μmol/L) | References |
| --- | --- | --- | --- | --- | --- | --- |
| 1 | c.-366A>G | - | 5’UTR | F (9m) | NA | Luksan (2010) |

**No category** (Splice site error)

| Variant No. | Nucleic acid | Amino acid | Location | Phenotype (onset-time) | NH3 (μmol/L) | References |
| --- | --- | --- | --- | --- | --- | --- |
| 15 | c.77+1G>A | - | Int 1 | N (4d) | NA | Matsuda (1997) |
|  |  |  |  | F (NA) | NA | Yamaguchi (2006) |
| 16 | c.77+1G>T | - | Int 1 | F (NA) | NA | Tuchman (1997) |
|  |  |  |  | F (NA) | 188 | This study |
| 17 | c.77+1G>C | - | Int 1 | N/F (1d) | 2,000 | Lu (2020) |
| 18 | c.77+2dupT | - | Int 1 | N (NA) | NA | Yamaguchi (2006) |
| 19 | c.77+3_77+6del | - | Int 1 | F (NA) | NA | Tuchman (2002) |
| 20 | c.77+4A>C | - | Int 1 | N (4d) | NA | Hoshide (1996) |
| 21 | c.77+5G>A | - | Int 1 | N (NA) | NA | Tuchman (1997) |
| 22 | c.77+5G>C | - | Int 1 | F (NA) | NA | Gobin-Limballe (2021) |
| 23 | c.78-3C>G | - | Int 1 | F (14m) | NA | Bisanzi (2002) |
| 24 | c.78-2A>G | - | Int 1 | L (3m) | 265 | Lu (2020) |
| 25 | c.78-1G>A | - | Int 1 | F (NA) | NA | Olga (2020) |
| 26 | c.78-1G>C | - | Int 1 | N (NA) | NA | Yamaguchi (2006) |
| 81 | c.216+1G>A | - | Int 2 | L (5m) | NA | Oppliger Leibundgut (1996b) |
|  |  |  |  | N (preneonatal) | NA | Kido (2021) |
| 82 | c.216+1G>T | - | Int 2 | L (NA) | 1,067 | Azevedo (2002) |
| 83 | c.217-2A>G | - | Int 2 | N (NA) | NA | Gobin-Limballe (2021) |
| 84 | c.217-1G>A | - | Int 2 | N (72h) | >3,000 | Tuchman (1992) |
| 124 | c.298+1G>A | - | Int 3 | N (7d) | NA | Garcia-Perez (1995b) |
| 125 | c.298+1G>T | - | Int 3 | L (NA) | NA | Yamaguchi (2006) |
| 126 | c.298+1G>C | - | Int 3 | F (NA) | NA | Martín-Hernández (2014) |
| 127 | c.298+2T>G | - | Int 3 | F (3.5y) | 234 | Lu (2020) |
| 128 | c.298+5G>C | - | Int 3 | F (1.3y) | 189 | Yamaguchi (2006) |
| 129 | c.298+1_298+5del | - | Int 3 | F (NA) | NA | Tuchman (1997) |
| 130 | c.299-8T>A | - | Int 3 | L (NA) | NA | Caldovic (2015) |
| 131 | c.299-7A>G | - | Int 3 | N (NA) | NA | Caldovic (2015) |
| 161 | c.386+1G>A | - | Int 4 | N (NA) | NA | Yamaguchi (2006) |
|  |  |  |  | F (NA) | NA | Martín-Hernández (2014) |
| 162 | c.386+1G>T | - | Int 4 | L (NA) | NA | Yamaguchi (2006) |
|  |  |  |  | L (19y)mo | 740 | Qin (2016) |
| 163 | c.386+1G>C | - | Int 4 | F (1y) | 352 | Ogino (2007) |
| 164 | c.386+2T>C | - | Int 4 | N (NA) | NA | Yamaguchi (2006) |
|  |  |  |  | F (NA) | NA | Martín-Hernández (2014) |
| 165 | c.386+4delT | - | Int 4 | L (2y) | 135 | Mohamed (2015) |
| 166 | c.386+5G>A | - | Int 4 | N (8d) | NA | Arranz (2007) |
| 167 | c.387-2A>C | - | Int 4 | N (NA) | NA | McCullough (2000) |
| 168 | c.387-2A>G | - | Int 4 | N (NA) | NA | McCullough (2000) |
| 169 | c.387-2A>T | - | Int 4 | N (NA) | NA | Carstens (1991) |
| 251 | c.540+1G>A | - | Int 5 | N (NA) | NA | Laróvere (2018) |
| 252 | c.540+1G>C | - | Int 5 | N (NA) | NA | Oppliger Leibundgut (1996b) |
| 253 | c.540+2T>A | - | Int 5 | N (NA) | NA | Yamaguchi (2006) |
| 254 | c.540+2T>G | - | Int 5 | NA | NA | Shchelochkov (2009) |
| 255 | c.540+2T>C | - | Int 5 | N (6d) | NA | Matsuura (1995) |
| 256 | c.540+5G>A | - | Int 5 | N (NA) | NA | Tuchman (1997) |
| 257 | c.540+265G>A | - | Int 5 | N (1d) | 235 | Ogino (2007) |
|  |  |  |  | F (1y) | 480 | Lu (2020) |
|  |  |  |  | F (3y) | 250 | Kumar (2021) |
|  |  |  |  | F (NA) | NA | Kumar (2021) |
|  |  |  |  | F (31y) | 96 | Kumar (2021) |
| 258 | c.541-2A>G | - | Int 5 | N (NA) | NA | Genet (2000) |
| 338 | c.663+1delG | - | Int 6 | NA | NA | Shchelochkov (2009) |
| 339 | c.663+1G>A | - | Int 6 | F (NA) | NA | Tuchman (1997) |
| 340 | c.663+1G>T | - | Int 6 | F (2m) | NA | Oppliger Leibundgut (1996b) |
| 341 | c.663+2T>C | - | Int 6 | F (NA) | NA | Tuchman (1997) |
| 342 | c.663+2dupT | - | Int 6 | F (NA) | NA | Tuchman (1995b) |
| 343 | c.664-1delG | - | Int 6 | F (NA) | NA | Tuchman (1997) |
| 344 | c.664-1G>A | - | Int 6 | N (NA) | NA | Tuchman (2002) |
|  |  |  |  | F (4y) | NA | Climent (2002) |
|  |  |  |  | F (6y) | 170 | Lu (2020) |
| 361 | c.717+1G>A | - | Int 7 | N (NA) | NA | Genet (2000) |
|  |  |  |  | F (24m) | 259 | Nguyen (2020) |
| 362 | c.717+1G>T | - | Int 7 | F (NA) | NA | Tuchman (2002) |
|  |  |  |  | F (6y) | 163 | Storkanova (2013) |
| 363 | c.717+2T>C | - | Int 7 | N (NA) | NA | Carstens (1991) |
|  |  |  |  | F (NA) | NA | Azevedo (2006) |
| 364 | c.717+3A>G | - | Int 7 | N (NA) | NA | Carstens (1991) |
|  |  |  |  | F (2.4y) | 344 | Choi (2015) |
| 365 | c.717+8_717+23del | - | Int 7 | N (NA) | NA | Calvas (1998) |
| 366 | c.718-2_731del | - | Int 7 | F (NA) | NA | Yamaguchi (2006) |
| 367 | c.718-2A>G | - | Int 7 | F (NA) | NA | Popowska (1999) |
| 368 | c.718-1G>A | - | Int 7 | F (2y) | 278 | Lu (2020) |
| 426 | c.867+1G>A | - | Int 8 | N (NA) | NA | Tuchman (1998) |
|  |  |  |  | F (2y) | NA | Hoshide (1993) |
| 427 | c.867+1G>T | - | Int 8 | N (NA) | NA | Oppliger Leibundgut (1996b) |
| 428 | c.867+1G>C | - | Int 8 | N (NA) | 600 | Li (2018) |
|  |  |  |  | N (NA) | 364 | Kido (2021) |
| 429 | c.867+1126A>G | - | Int 8 | L (6m) | NA | Engel (2008) |
| 430 | c.868-3T>C | - | Int 8 | L (17y) | 258 | Lee (2014) |
|  |  |  |  | L (20y) | 40 | Lee (2014) |
| 431 | c.868-1G>C | - | Int 8 | F (4y) | >500 | Lu (2020) |
| 493 | c.1005+1G>A | - | Int 9 | F (2y) | 364 | Shao (2017) |
| 494 | c.1005+1G>T | - | Int 9 | N (NA) | NA | Tuchman (1997) |
| 495 | c.1005+2T>C | - | Int 9 | N (NA) | NA | Tuchman (2002) |
| 496 | c.1005+1091C>G | - | Int 9 | L (11m) | NA | Engel (2008) |
| 497 | c.1006-1G>A | - | Int 9 | N (NA) | NA | Gyato (2004) |
| 498 | c.1006-3C>G | - | Int 9 | L (16m) | NA | Climent (2002) |

N: neonatal-onset, L: late-onset, F: female, NA: not available, mo: mosaicism
